# Validating the Core Set for Vocational Rehabilitation in a Population of Cancer Survivors: A Cross-Sectional Study

**DOI:** 10.1007/s10926-024-10252-5

**Published:** 2024-12-11

**Authors:** Sara Paltrinieri, Martina Pellegrini, Stefania Costi, Stefania Fugazzaro, Massimo Vicentini, Pamela Mancuso, Paolo Giorgi Rossi

**Affiliations:** 1 Research and EBP Unit, Health Professions Department, Azienda USL-IRCCS Di Reggio Emilia, Reggio Emilia, Italy; 2 Public Health Sciences PhD Program, Department of Clinical Sciences and Community Health, University of Milan, Milan, Italy; 3 Scientific Directorate, Azienda USL-IRCCS Di Reggio Emilia, Reggio Emilia, Italy; 4 Department of Surgery, Medicine, Dentistry and Morphological Sciences, University of Modena and Reggio Emilia, Modena, Italy; 5 Physical Medicine and Rehabilitation Unit, Azienda USL-IRCCS Di Reggio Emilia, Reggio Emilia, Italy; 6 Epidemiology Unit, Azienda USL-IRCCS Di Reggio Emilia, Reggio Emilia, Italy

**Keywords:** Cancer survivors, Vocational rehabilitation, International Classification of Functioning, Disability and Health, Return to work

## Abstract

**Purpose:**

The Core Set for Vocational Rehabilitation (CS-VR), a checklist based on the International Classification of Functioning, Disability and Health (ICF), captures the work functioning of individuals requiring VR. By listening to cancer survivors’ experiences and stakeholders’ perspectives, the CS-VR-Onco of 85 ICF-based categories was obtained. The aim of this study was to assess the concurrent validity of the CS-VR-Onco by measuring this tool’s ability to detect differences among cancer survivors in terms of perceived return to work (RTW)-related difficulties.

**Methods:**

A sample of 300 working-age individuals with a first diagnosis of cancer was selected through the local Cancer Registry. Of these 300, those employed individuals who had returned to work were deemed eligible. Through a guided interview, participants reported and described their perceived RTW-related difficulties using the terminology of the CS-VR-Onco. Frequencies and means were used to compare the results of (a) cancer survivors who reported having had difficulties with those who had not, and (b) cancer survivors who had undergone chemotherapy (CT) with those who had not.

**Results:**

Of the 104 respondents, 35 cancer survivors (Group 1) reported having had RTW-related difficulties and CS-VR-Onco-described problems, while 54 reported no difficulties but did highlight some problems (Group 2), and 15 reported neither difficulties nor problems (Group 3). The categories of the CS-VR-Onco that were prioritized were similar across groups, but Group 1 had higher frequencies than did Group 2 + 3 in 69 categories out of 85. In the second comparison, 40 cancer survivors who had undergone CT had higher frequencies than did 64 cancer survivors who had not undergone CT, but this trend was not applicable to 23 categories of the CS-VR-Onco. Seven categories were not reported as problems by all participants.

**Conclusion:**

The CS-VR-Onco identified more problems in cancer survivors who reported RTW-related difficulties and differences between cancer survivors who had undergone CT and those who had not. These results contribute to assessing the preliminary validity of the tool.

**Supplementary Information:**

The online version contains supplementary material available at 10.1007/s10926-024-10252-5.

## Introduction

Return to work (RTW) is one of the rehabilitation goals for cancer survivors to restore their social integration and involvement in the community [1]. Work reintegration positively affects wellbeing and quality of life [2] by minimizing cancer-related physical, psychological, and social consequences. Furthermore, for some patients, work may be essential to maintaining financial independence [3]. However, the employment rate of cancer survivors varies, and RTW is not always achieved [4]. Work reintegration and maintaining employment are major issues for society as well, given that patients of working age accounted for 47% of the estimated 19 million new cancer diagnoses in 2020 [5]. Therefore, surveillance of the working conditions of this population is required [6] in order to overcome barriers that could impact the RTW process and to address potential gaps in the provision of care.

Vocational rehabilitation (VR) can support the RTW process of individuals with work-related difficulties. VR is defined as “a multi-professional evidence-based approach that is provided in different settings, services, and activities to working-age individuals with health-related impairments, limitations, or restrictions with work functioning and whose primary aim is to optimize work participation” [7]. The ultimate goal of VR interventions is to improve work functioning through a multidisciplinary approach by matching the needs of workers with the needs of the workplace [8], which seems to facilitate RTW [9]. Nonetheless, a careful evaluation of potential candidates to VR is suggested to maximize the benefits of the intervention and to distribute resources appropriately [10].

The International Classification of Functioning, Disability and Health (ICF) was developed as a suitable and comprehensive theoretical framework for this purpose [11, 12]. The ICF has a hierarchical structure of which different levels are components, chapters, and categories. The four components are Body Functions (BF), Body Structures (BS), Activity and Participation (AP), and Environmental Factors (EF). Each component is divided into chapters, and each chapter comprises several categories. There is an illustration of the ICF framework as follows:

**Table:** 

Component	Body Functions
Chapter	b2 Sensory function and pain
Category	b280 Sensation of pain

Given that the ICF contains 1465 alphanumeric codes to describe health status, the ICF Research Branch identified the need to create Core Sets, i.e., shorter ICF-based checklists for specific diseases or conditions, to facilitate their applicability in clinical practice.

The Core Set for Vocational Rehabilitation (CS-VR) is a 90-category ICF-based checklist aimed to capture work functioning and to guide the multidisciplinary assessment of individuals requiring VR.

The CS-VR is based on the common and unified language of the ICF framework, which facilitates communication between healthcare professionals and the intersectoral and multidisciplinary coordination of services [12]. The CS-VR is well structured as it provides detailed information about the work issues that a patient may experience during his/her return to work process. For this reason, it is valuable to plan a VR program. The CS-VR was developed through a multiphase decision-making and consensus process involving clinicians, patients, and international experts [13–16]. None of the patients involved in the CS-VR development process had cancer [15]. Starting from the CS-VR, an assessment tool was developed to evaluate the work functioning of individuals in need of VR [17], but in this case as well, none of patients involved had cancer. Moreover, this latter tool has not yet been validated in Italian. Thus, as a first approach to the oncology setting, the CS-VR seemed to be the ideal checklist from which to start to describe the work functioning of cancer survivors as well as to map the barriers and facilitators in this area. A formal linguistic and cross-cultural adaptation of the CS-VR in Italian was not necessary since it had already been performed during the development process of the ICF [18]. Therefore, an exploratory mixed-method study was conducted to obtain a CS-VR for cancer survivors in need of VR [19]. We first adapted the CS-VR by listening to cancer survivors talk about their RTW experiences during five focus groups [19]. We obtained the preliminary version of the CS-VR-Onco by linking the experiences of cancer survivors to the ICF categories. Then, during a group interview, we integrated the point of view of the stakeholders of the RTW process by asking them whether the preliminary version was complete. Stakeholders reported that CS-VR-Onco was in-depth in its content and in line with their professional experience; no one felt that any category was missing. Through this first qualitative phase, we obtained the CS-VR-Onco. The CS-VR-Onco is composed of 85 categories based on the ICF: 26 for BF, 33 for AP, and 26 for EF. The structure of the CS-VR-Onco is illustrated as follows:

**Table:** 

Components	Body functions (BF)	Activity and Participation (AP)	Environmental Factors (EF)
No. of chapters	4	8	5
List of chapters	Mental functions; Sensory functions and pain; Exercise and tolerance functions; Other functions	Learning and applying knowledge; General tasks and demands; Communication; Mobility; Self-care; Interpersonal interactions and relationships; Major life areas; Community, social and civic life	Products and technology; Natural environment and human-made changes to environment; Support and relationships; Attitudes; Services, systems, and policies
Number of categories	26	33	26

Validation of an ICF-based Core Set is an important step towards understanding whether its categories capture the construct they should describe [20]. A recent scoping review searched for articles describing the validation of Core Sets [21]; two studies were found, one describing the validation of the original CS-VR from the perspective of individuals with spinal cord injury [22], and the other from the perspective of physical therapists working in the VR setting [23].

Since the development of the CS-VR-Onco, no study on the validity of this tool in the population of interest has been conducted. The aim of this study was, therefore, to preliminarily validate the CS-VR-Onco, in the absence of a gold standard, by measuring its internal validity and external validity in investigating cancer survivors’ perceived RTW-related difficulties.

## Methods

### Study Design and Rationale

This cross-sectional study was conducted in the province of Reggio Emilia (pop. 529,000), an area located in the Emilia-Romagna region in Northern Italy. The main hospital is located in the city of Reggio Emilia, and five smaller hospitals are located throughout the province. Potential participants in this study were selected through the accredited Cancer Registry of the province of Reggio Emilia, which registers all malignant tumors diagnosed in the resident population, and were contacted by letter between October and December 2021. Data were collected between November 2021 and March 2022.

We had no gold standard to validate the CS-VR-Onco. Although there was another tool that evaluates work functioning of individuals in need of VR [17], it involved patients who had not had cancer; in addition, it has not yet been translated and nor undergone cross-cultural validation in Italian. Furthermore, the CS-VR-Onco is designed to map and describe the possible work issues to help plan a VR program. For this reason, it is not possible to collapse the information into a yes-or-no diagnostic criterion or to summarize the results in an overall score. Indeed, there are no simple gold standard and accuracy measures that can be used to validate CS-VR-Onco since it does not produce one overall score. Therefore, we propose a concurrent validation of the CS-VR-Onco based on two a priori hyphoteses supported by the logic of the tool and by evidence from the literature:

1. To assess internal validity, we chose to group the participants based on their perceived difficulty in the RTW process (yes/no difficulty) and on the description of those difficulties in the components, chapters, and categories of the CS-VR-Onco.
2. To assess external validity, we chose to distinguish between participants based on the variable *chemotherapy* (CT). We formulated this hypothesis in light of the results of previous studies [4, 24, 25] that found that cancer survivors who had undergone CT seemed to have more RTW-related difficulties. Thus, we expected that participants who had undergone CT would experience greater problems in the RTW process than would those who had not undergone CT.

The preliminary validation of the CS-VR-Onco was considered as satisfied if the instrument was able to show differences between the identified groups.

## Participants and Study Size

Participation was restricted to individuals (a) with a first diagnosis of infiltrating malignant tumor in 2018 who were, (b) in working age at diagnosis (20–60 years), and were (c) employed at diagnosis or who, (d) had returned to work. Individuals diagnosed with non-melanoma skin cancer, comorbidities, or linguistic barriers that restricted communication were excluded. To achieve the aim of the study, recruitment took place approximately 3–4 years after diagnosis because cancer survivors likely had returned to work by then and were, therefore, in a position to describe their experiences.

In 2018, there were 4459 first diagnoses of infiltrating malignant tumors in the province. After excluding individuals who had died before the study began, those affected by non-melanoma skin cancer, and those who were not in the working-age population, a sample of 758 eligible individuals was reached.

A priori, we decided to interview a cohort of at least 100 participants, including cancer survivors whose diagnosis was associated with a greater likelihood of RTW difficulties, as reported in the cross-sectional study by Paltrinieri S. et al. 2020 [26]. To reach this number, we contacted a random sample of 300 individuals from those potentially eligible (n.758). A simple randomization using a random number draw using Stata16 software was done. The number of individuals of the random sample was determined by considering that, in the previous study [26], half of the eligible cancer survivors who were contacted for participation were interviewed. In addition, considering that the eligibility criteria of the two studies did not completely overlap (in our study, only cancer survivors who had returned to work were eligible), we decided to increase the number of individuals to be contacted.

Thus, a sample of 300 potentially eligible patients were contacted by letter and informed about study aims and methods. The letter stated that the principal investigator of the study would contact each potential participant by telephone to give any further information about study participation. Since data regarding employment status are not routinely collected by the Cancer Registry of the province of Reggio Emilia, eligibility criteria (c) and (d) were assessed directly during the first phone contact. If these criteria were satisfied and if the patient agreed to participate, an appointment was scheduled at the hospital, at home, or online to conduct the interview. Informed consent was collected from all participants before the beginning of the interview.

## Description of the Tool and Interviews

This cross-sectional study is based on a guided interview composed of 231 questions. The interview took inspiration from those employed in previous studies to collect data concerning the RTW of cancer survivors in our context [19, 26]. The interview was translated into English (Supplementary Information 1-SI1). Participants were first asked questions regarding sociodemographic data (n. 5 questions), cancer-related factors (n. 7 questions), and work-related factors (n. 17 questions). Then, participants were asked if they had perceived any difficulty in the RTW process through one question with a dichotomous answer. They were then asked which of the 85 categories of the CS-VR-Onco corresponded with their RTW-related difficulties and to describe them (n. 170 questions). In addition, participants were asked whether they considered the 26 categories of the EF component as barriers or as facilitators (n. 26 questions). Although this study considered the 85 categories of the CS-VR-Onco as reported or not reported (dichotomous answer), healthcare professionals in the clinical setting can score these categories from 0 to 4 to describe the level of disability. Finally, information regarding the intelligibility of the questionnaire, the length of its administration, and any missing concepts were collected (n. 5 questions).

The interviews were conducted either by S.P. or by M.P. who are both occupational therapists and researchers. The two interviewers conducted the first ten interviews together to clarify any doubts and to be aligned in the conduction of the interviews. Furthermore, the interviewers referred to the ICF framework definitions to help participants who did not understand the meaning of the category by using also examples identified a priori. The interviews were not audio-recorded.

## Outcomes and Variables for Validation

The outcomes were the frequencies of categories included in the CS-VR-Onco reported by participants. Regarding the BF and AP components, the categories were the reported RTW-related difficulties, while for the EF, the categories could influence the RTW process as barriers, facilitators, or both.

To describe internal validity, frequencies were compared between groups of cancer survivors:

- Group 1—participants who perceived RTW-related difficulties, which were described through the components, the chapters, and the categories of the CS-VR-Onco.
- Group 2—participants who reported not having had any RTW-related difficulties but who nevertheless reported problems during this process only as described through the components, the chapters, and the categories of the CS-VR-Onco.
- Group 3—participants who did not perceive any difficulties in RTW and who also did not report any problems during this process as described in the CS-VR-Onco.

Subsequently, we merged Group 2 and Group 3 to compare frequencies between the participants who perceived RTW-related difficulties and CS-VR-Onco-described problems (Group 1) and those who did not (Groups 2 + 3).

To describe external validity, frequencies were compared between the group of patients who had undergone CT and those who had not. Participants were categorized into these groups according to their answer in the guided interview.

## Statistical Analysis

Regarding internal validity and external validity, we counted the number of categories of the BF and AP components of the CS-VR-Onco that each group described as a problem as well as the number of categories of the EF component that influenced the RTW process as either a barrier, a facilitator, or both. We calculated the total number of categories reported as a problem and the corresponding percentages of the total number of possible answers, the range of categories, the average number of categories, and the number of participants with the corresponding number of categories reported.

## Results

**Fig. 1:**
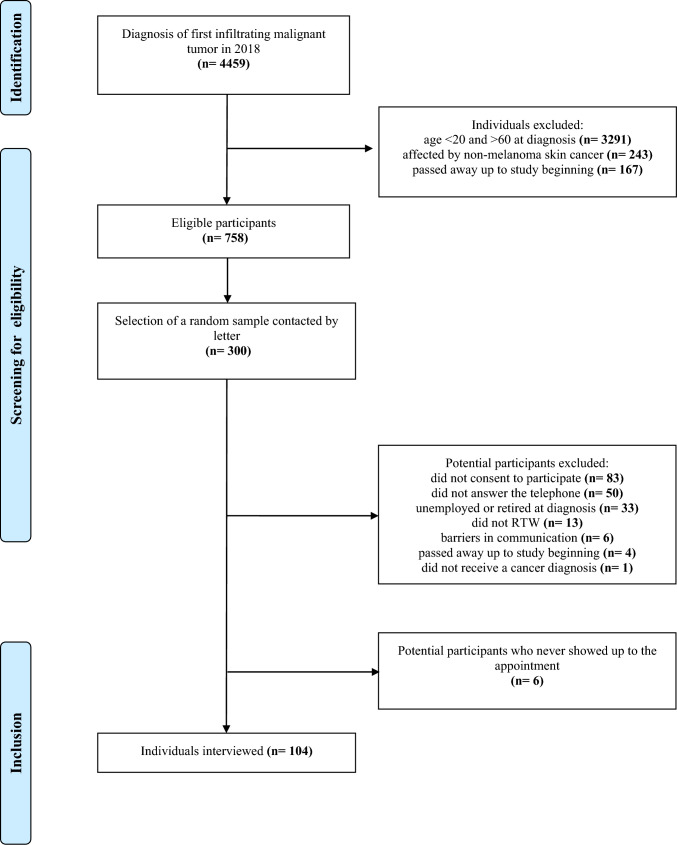
Flowchart of recruitment from the Cancer Registry of the province of Reggio Emilia

Of the 300 potentially eligible participants who were sent the information letter about this study, 196 individuals were excluded. The reasons for exclusion included not verbally consenting to participate, not answering the telephone, being unemployed, or retired at diagnosis, not having RTW, having communication barriers, having died before the start of the study, and not having had a cancer diagnosis. Moreover, six individuals never showed up to the appointment. Thus, we interviewed 104 cancer survivors (Fig. 1).

Table 1 reports the sociodemographic information and the cancer-related and work-related factors by groups of participants. Of the total sample, the majority of participants were female (58.7%), married (67.3%), had a high education level (80.8%), and had at least one child (78.9%). The most represented tumor types were breast cancer (28.8%), hematological malignancies (13.5%), skin cancer (9.6%), and thyroid cancer (9.6%). Most of the participants had not undergone CT (61.5%), whereas almost all had undergone surgery (84.6%). Most of the participants were employees (79.8%), employed in the private sector (63.5%), with permanent contracts (79.8%) and with full-time work schedules (81.7%).

**Table 1:** Sociodemographic data, cancer-related factors, and work-related factors by groups of participants

			Total	Perceived RTW-related difficulties and CS-VR-Onco-described problems			Type of treatment	
				Group 1	Group 2	Group 3	Group CT	Group NoCT
			n	n (%)	n (%)	n (%)	n (%)	n (%)
			104	35 (33.7)	54 (51.9)	15 (14.4)	40 (38.5)	64 (61.5)
Sociodemographic data	Sex	Male	43 (41.3)	10 (23.3)	22 (51.2)	11 (25.6)	12 (27.9)	31 (72.1)
		Female	61 (58.7)	25 (41)	32 (52.5)	4 (6.6)	28 (45.9)	33 (54.1)
	Age at diagnosis (years)	≤ 50	51 (49.0)	18 (35.3)	25 (49.0)	8 (15.7)	20 (39.2)	31 (60.8)
		> 50	53 (51.0)	17 (32.1)	29 (54.7)	7 (13.2)	20 (37.7)	33 (62.3)
	Marital status	Married/cohabitant	70 (67.3)	20 (28.6)	38 (54.3)	12 (17.1)	25 (35.7)	45 (64.3)
		Single	16 (15.4)	6 (37.5)	9 (56.3)	1 (6.3)	8 (50.0)	8 (50.0)
		Widowed	1 (1.0)	1 (100.0)	0 (0.0)	0 (0.0)	0 (0.0)	1 (100.0)
		Divorced	11 (10.6)	4 (36.4)	5 (45.5)	2 (18.2)	3 (27.3)	8 (72.7)
		Separated	6 (5.8)	4 (66.7)	2 (33.3)	0 (0.0)	4 (66.7)	2 (33.3)
	Education level	Primary school	2 (1.9)	1 (50.0)	1 (50.0)	0 (0.0)	2 (100.0)	0 (0.0)
		Middle school	17 (16.3)	5 (29.4)	9 (52.9)	3 (17.6)	6 (35.3)	11 (64.7)
		High school	52 (50.0)	14 (26.9)	32 (61.5)	6 (11.5)	22 (42.3)	31 (59.6)
		University degree	32 (30.8)	15 (46.9)	12 (37.5)	5 (15.6)	10 (31.3)	22 (68.8)
		Other	1 (1.0)	0 (0.0)	0 (0.0)	1 (100.0)	0 (0.0)	1 (100.0)
	Children	0	22 (21.1)	5 (22.7)	13 (59.1)	4 (18.2)	9 (40.9)	13 (59.1)
		1	34 (32.7)	14 (41.2)	14 (41.2)	6 (17.6)	14 (41.2)	20 (58.8)
		≥ 2	48 (46.2)	16 (33.3)	27 (56.3)	5 (10.4)	17 (35.4)	31 (64.6)
Cancer-related factors	Tumor type	Breast	30 (28.8)	14 (46.7)	15 (50.0)	1 (3.3)	18 (60.0)	12 (40.0)
		Hematological	14 (13.5)	4 (28.6)	7 (50.0)	3 (21.4)	11 (78.6)	3 (21.4)
		Skin	10 (9.6)	2 (20.0)	5 (50.0)	3 (30.0)	0 (0.0)	10 (100.0)
		Thyroid	10 (9.6)	4 (40.0)	5 (50.0)	1 (10.0)	0 (0.0)	10 (100.0)
		Female organs	8 (7.7)	2 (25.0)	5 (62.5)	1 (12.5)	3 (37.5)	5 (62.5)
		Prostate	7 (6.7)	1 (14.3)	4 (57.1)	2 (28.6)	1 (14.3)	6 (85.7)
		Bladder	7 (6.7)	2 (28.6)	3 (42.9)	2 (28.6)	1 (14.3)	6 (85.7)
		Colon	6 (5.8)	3 (50.0)	3 (50.0)	0 (0.0)	2 (33.3)	4 (66.7)
		Head neck	3 (2.9)	1 (33.3)	2 (66.7)	0 (0.0)	2 (66.7)	1 (33.3)
		Pancreas	2 (1.9)	1 (50.0)	0 (0.0)	1 (50.0)	1 (50.0)	1 (50.0)
		Brain	2 (1.9)	1 (50.0)	0 (0.0)	1 (50.0)	0 (0.0)	2 (100.0)
		Male organs	1 (1.0)	0 (0.0)	1 (100.0)	0 (0.0)	0 (0.0)	1 (100.0)
		Sarcoma	1 (1.0)	0 (0.0)	1 (100.0)	0 (0.0)	0 (0.0)	1 (100.0)
		Mesothelioma	1 (1.0)	0 (0.0)	1 (100.0)	0 (0.0)	0 (0.0)	1 (100.0)
		Liver	1 (1.0)	0 (0.0)	1 (100.0)	0 (0.0)	0 (0.0)	1 (100.0)
		Lung	1 (1.0)	0 (0.0)	1 (100.0)	0 (0.0)	1 (100.0)	0 (0.0)
	Chemotherapy	No	64 (61.5)	17 (26.6)	33 (51.6)	14 (21.9)	–	–
		Yes	40 (38.5)	18 (45.0)	21 (52.5)	1 (2.5)	–	–
	Surgery	Yes	88 (84.6)	29 (33.0)	48 (54.5)	11 (12.5)	29 (33.0)	59 (67.0)
	Axillary lymph node	Yes	26 (25)	11 (42.3)	12 (46.2)	3 (11.5)	14 (53.8)	12 (46.1)
	Radiotherapy	Yes	38 (36.5)	19 (50.0)	17 (44.7)	2 (5.3)	24 (63.2)	14 (36.8)
	Hormone therapy	Yes	25 (24.0)	11 (44.0)	13 (52.0)	1 (4.0)	12 (48.0)	13 (52.0)
	Bone marrow transplant	Yes	4 (3.8)	1 (25.0)	3 (75.0)	0 (0.0)	4 (100.0)	0 (0.0)
Work-related factors	Occupational status	Employee	83 (79.8)	28 (33.7)	43 (51.8)	12 (14.5)	32 (38.6)	51 (61.4)
		Self-employed	18 (17.3)	6 (33.3)	10 (55.6)	2 (11.1)	7 (38.9)	11 (61.1)
		Other	3 (2.9)	1 (33.3)	1 (33.3)	1 (33.3)	1 (33.3)	2 (66.7)
	Type of company	Private sector	66 (63.5)	24 (36.4)	33 (50.0)	9 (13.6)	26 (39.4)	40 (60.6)
		Public sector	30 (28.8)	10 (33.3)	16 (53.3)	4 (13.3)	12 (40.0)	18 (60.0)
		I do not know	8 (7.7)	1 (12.5)	5 (62.5)	2 (25.0)	2 (25.0)	6 (75.0)
	Type of contract	Permanent contract	83 (79.8)	28 (33.7)	43 (51.8)	12 (14.5)	33 (39.3)	50 (59.5)
		Fixed-term contract	3 (2.9)	1 (33.3)	1 (33.3)	1 (33.3)	0 (0.0)	3 (100.0)
		Self-employed	18 (17.3)	6 (33.3)	10 (55.6)	2 (11.1)	7 (41.2)	11 (64.7)
	Work schedule	Full-time	85 (81.7)	29 (34.1)	43 (50.6)	13 (15.3)	35 (41.2)	50 (58.8)
		Part-time	18 (17.3)	6 (33.3)	10 (55.6)	2 (11.1)	5 (27.8)	13 (72.2)
		Other	1 (1.0)	0 (0.0)	1 (100.0)	0 (0.0)	0 (0.0)	1 (100.0)
	Shift worker	No	98 (94.2)	33 (33.7)	51 (52.0)	14 (14.3)	38 (38.8)	60 (61.2)
		Yes	6 (5.8)	2 (33.3)	3 (50.0)	1 (16.7)	2 (33.3)	4 (66.7)
	Work in the evening	No	84 (80.8)	25 (29.8)	48 (57.1)	11 (13.1)	34 (40.5)	50 (59.5)
		Yes	20 (19.2)	10 (50)	6 (30.0)	4 (20.0)	6 (30.0)	14 (70.0)
	Work at night	No	98 (94.2)	32 (32.7)	52 (53.1)	14 (14.3)	38 (38.8)	60 (61.2)
		Yes	6 (5.8)	3 (50.0)	2 (33.3)	1 (16.7)	2 (33.3)	4 (66.7)
	Work on the weekend	No	54 (51.9)	15 (27.8)	31 (57.4)	8 (14.8)	22 (40.7)	32 (59.3)
		Yes	50 (48.1)	20 (40.0)	23 (46.0)	7 (14.0)	18 (36.0)	32 (64.0)
	Flexible work schedule	No	44 (42.3)	14 (31.8)	21 (47.7)	9 (20.5)	16 (36.4)	28 (63.6)
		Yes	60 (57.7)	21 (35.0)	33 (55.0)	6 (10.0)	24 (40.0)	36 (60.0)
	Flexible work tasks	No	52 (50.0)	15 (28.8)	27 (51.9)	10 (19.2)	18 (34.6)	34 (65.4)
		Yes	52 (50.0)	20 (38.5)	27 (51.9)	5 (9.6)	22 (42.3)	30 (57.7)
	Number of worker in the company	Alone	8 (7.7)	2 (25.0)	5 (62.5)	1 (12.5)	2 (25.0)	6 (75.0)
		< 10	25 (24.0)	11 (44.0)	11 (44.0)	3 (12.0)	13 (52.0)	12 (48.0)
		10–49	20 (19.2)	6 (30.0)	12 (60.0)	2 (10.0)	8 (40.0)	12 (60.0)
		50–249	21 (20.2)	7 (33.3)	10 (47.6)	4 (19.0)	8 (38.1)	13 (61.9)
		> 249	30 (28.8)	9 (30.0)	16 (53.3)	5 (16.7)	9 (30.0)	21 (70.0)
	Work experience (years)	< 1	6 (5.8)	1 (16.7)	4 (66.7)	1 (16.7)	4 (66.7)	2 (33.3)
		1–4	18 (17.3)	4 (22.2)	10 (55.6)	4 (22.2)	4 (22.2)	14 (77.8)
		5–10	6 (5.8)	1 (16.7)	5 (83.3)	0 (0.0)	4 (40.0)	6 (60.0)
		> 10	74 (71.2)	29 (39.2)	35 (47.3)	10 (13.5)	28 (37.8)	42 (56.8)
	Psychologically demanding job	no	41 (39.4)	9 (22.0)	21 (51.2)	11 (26.8)	17 (41.5)	24 (58.5)
		yes	47 (45.2)	21 (44.7)	24 (51.1)	2 (4.3)	17 (36.2)	30 (63.8)
		sometimes	16 (15.4)	5 (31.3)	9 (56.3)	2 (12.5)	6 (37.5)	10 (62.5)
	Physically demanding job	no	59 (56.7)	16 (27.1)	31 (52.5)	12 (20.3)	23 (39.0)	36 (61.0)
		yes	28 (26.9)	14 (50.0)	12 (42.9)	2 (7.1)	10 (35.7)	18 (64.3)
		sometimes	17 (16.3)	5 (29.4)	11 (64.7)	1 (5.9)	7 (41.2)	10 (58.8)

Supplementary Information 2 (SI2) reports the type of accommodations by groups of participants. Of the total sample, the majority of participants did not report any accommodations (55.8%).

The interviews lasted an average of 47 min (range 15–180), and all the participants answered all of the questions; as such, there were no missing values. Participants reported an average of 7.7 RTW-related difficulties in both the BF and AP components and 4.6 factors affecting the RTW process in the EF component (data not shown).

### Internal validity

Regarding internal validity, Groups 1, 2, and 3 included 35, 54, and 15 participants, respectively. Therefore, Group 2 + 3 comprised 69 participants. Supplementary Information 3 (SI3) presents the categories and the components of BF, AP, and EF reported by the three groups of participants. Group 1 reported more categories as problems (BF = 28.1%; AP = 18%) than did Group 2 (BF = 15.7%; AP = 6.2%). As for EF, Group 1 reported more categories as barriers (26.5%) than did Group 2 (21.7%), while Group 3 reported more facilitators (92.6%) than did Group 1 (71.1%) and Group 2 (74.4%).

Group 1 reported an average of 7.3 and 5.9 problems in the BF and AP components, respectively, and 6.9 factors influencing the RTW process in the EF component. Group 2 + 3 reported an average of 3.2 and 1.6 of problems per participant in the BF and AP components, respectively, and 3.4 factors influencing the RTW process per participant in the EF component (data not shown). The number of cancer survivors who reported five or more problems was greater in Group 1 (BF = 26; AP = 20) than in Group 2 + 3 (BF = 22; AP = 5).

Supplementary Information 4 (SI4) presents the categories and the chapters of the BF and AP components. Regarding the BF component, Group 1 showed the highest percentages of problems in all chapters as compared to Group 2 + 3, specifically in *b4 Exercise and tolerance functions* (47.1%), *b1 Mental functions* (38.1%), and *b2 Sensory functions and pain* (20%). These were also the most frequently identified problems by Group 2 + 3, but with lower percentages (b4 = 21.7%; b1 = 16.6%). Regarding the AP component, Group 1 showed the highest percentages of problems in all chapters as compared to Group 2 + 3, in particular concerning *d2 General tasks and demands* (30%), *d8 Major life areas* (22.9%), and *d7 Interpersonal interactions and relationships* (20.6%); the latter chapter was not a major issue for Group 2 + 3 (d7 = 2.9%).

**Fig. 2:**
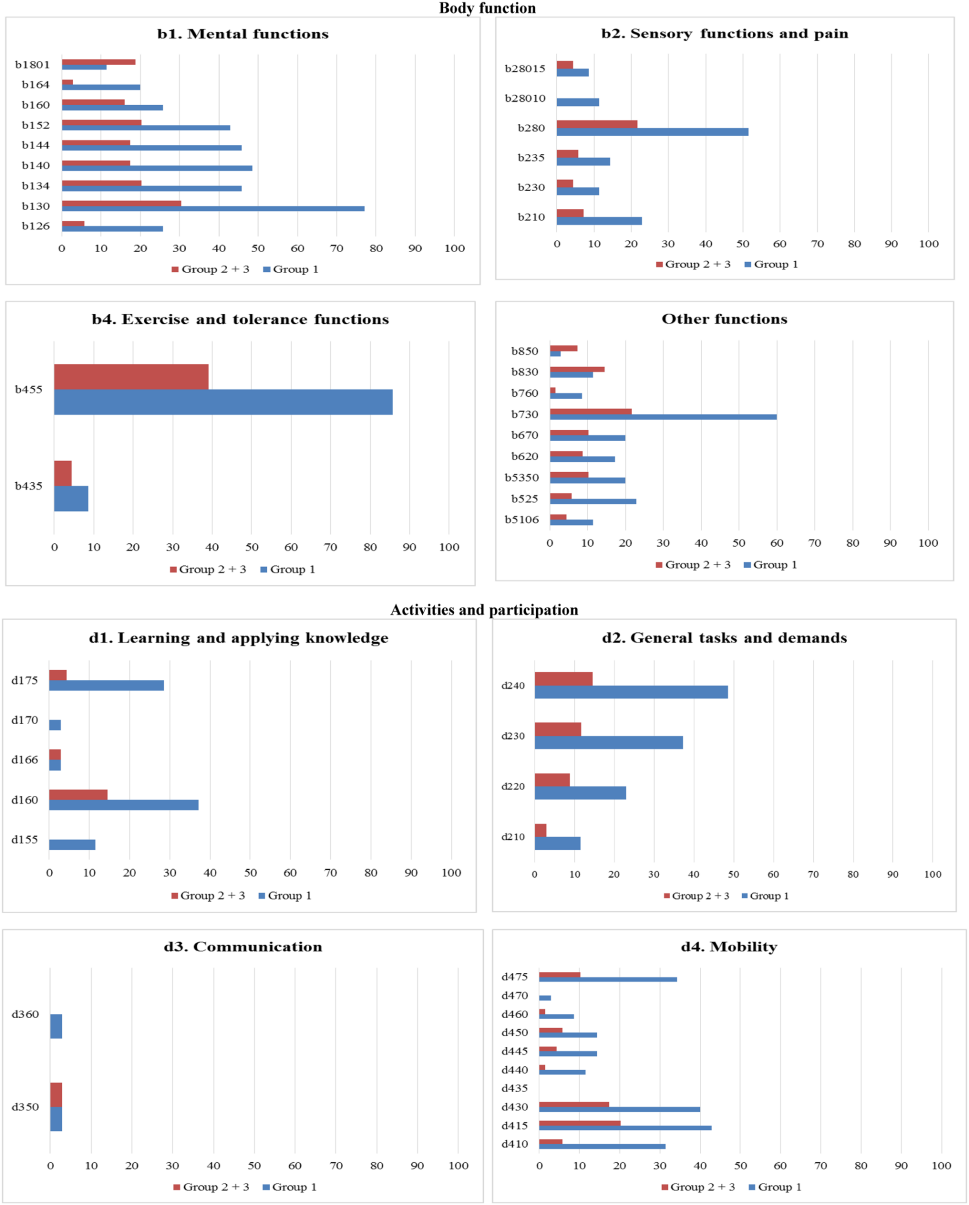

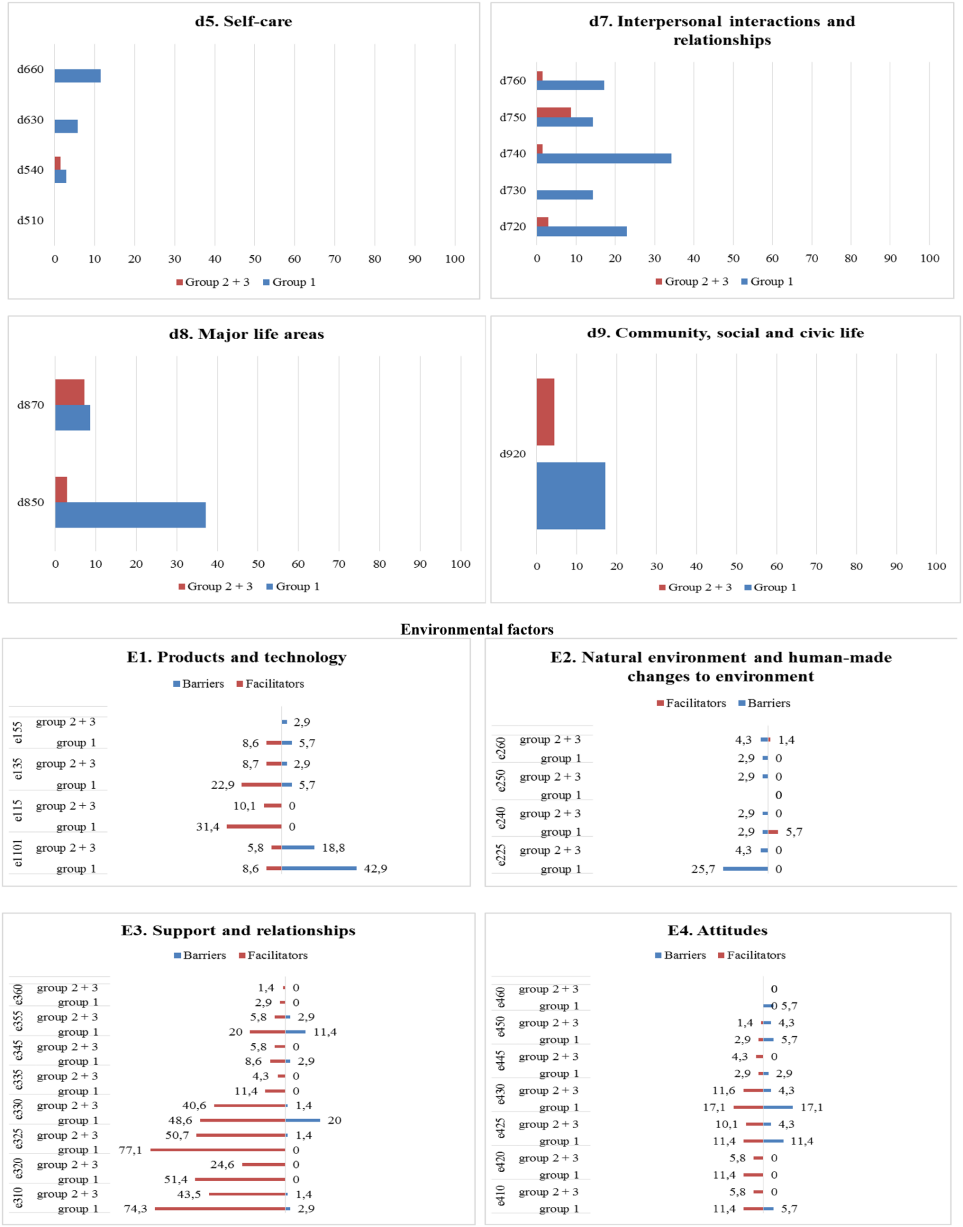

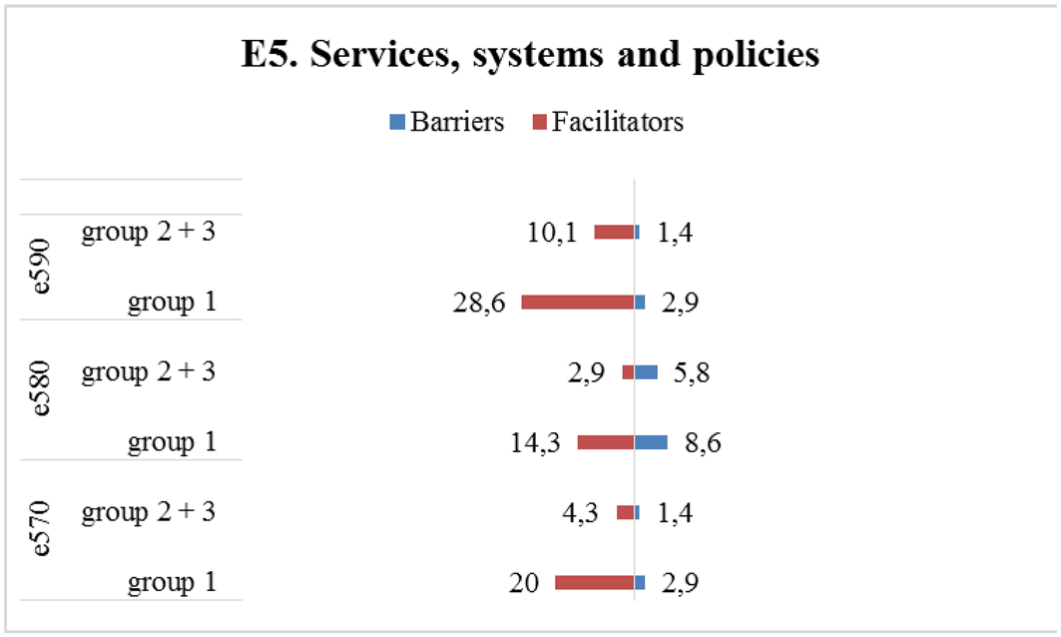
Description of categories **BF component.** b1. Mental functions: b126. Temperament and personality functions, b130. Energy and drive functions, b134. Sleep functions, b140. Attention functions, b144. Memory functions, b152. Emotional functions, b160. Thought functions, b164. Higher-level cognitive functions, b1801. Body image. b2. Sensory function and pain: b210. Seeing functions, b230. Hearing functions, b235. Vestibular functions, b280. Sensation of pain, b28010. Pain in head and neck, b28015. Pain in lower limb; b4. Exercise and tolerance functions: b435. Immunological system functions, b455. Exercise tolerance functions; Other functions: b5106. Functions of expelling the contents of the stomach, esophagus or pharynx, b525. Defecation functions, b5350. Sensation of nausea, b620. Urination functions, b670. Sensations associated with genital and reproductive functions, b730. Muscle power functions, b760. Control of voluntary movement functions, b830. Other functions of the skin, b850. Functions of hair. **AP component.** d1. Learning and applying knowledge: d155. Acquiring skills, d160. Focusing attention, d166. Reading, d170. Writing, d175. Solving problems; d2. General tasks and demands: d210. Undertaking a single task, d220. Undertaking multiple tasks, d230. Carrying out daily routine, d240. Handling stress and other psychological demands; d3. Communication: d350. Conversation, d360. Using communication devices and techniques; d4. Mobility: d410. Changing basic body position, d415. Maintaining a body position, d430. Lifting and carrying objects, d435. Moving objects with lower extremities, d440. Fine hand use, d445. Hand and arm use, d450. Walking, d460. Moving around in different locations, d470. Using transportation, d475. Driving; d5. Self-care and Domestic life: d510. Washing oneself, d540. Dressing, d630. Preparing meals, d660. Assisting others; d7. Interpersonal interactions and relationships: d720. Complex interpersonal interactions, d730. Relating with strangers, d740. Formal relationships, d750. Informal social relationships, d760. Family relationships; d8. Major life areas: d850. Remunerative employment, d870. Economic self-sufficiency; d9. Community, social and civic life: d920. Recreation and leisure. **EF component.** e1. Products and technology: e1101. Drugs, e115. Products and technology for personal use in daily living, e135. Products and technology for employment e155. Design, construction and building products and technology of buildings for private use; e2. Natural environment and human-made changes to environment, e225. Climate, e240. Light, e250. Sound, e260. Air quality; e3. Support and relationships: e310. Immediate family, e320. Friends, e325. Acquaintances, peers, colleagues, neighbours and community members, e330. People in positions of authority, e335. People in subordinate positions, e345. Strangers, e355. Health professionals, e360 Other professionals; e4. Attitudes: e410. Individual attitudes of immediate family members, e420. Individual attitudes of friends, e425. Individual attitudes of acquaintances, peers, colleagues, neighbors and community members, e430. Individual attitudes of people in positions of authority, e445. Individual attitudes of strangers, e450. Individual attitudes of health professionals, e460. Societal attitudes; e5. Services, systems, and policies: e570. Social security services, systems, and policies, e580. Health services, systems, and policies e590. Labour and employment services, systems, and policies. Percentages of categories of the BF, AP, and EF components reported by participants in Group 1 and in Group 2 + 3

Figure 2 shows the percentages of the categories of the BF and AP components reported by participants in Group 1 and in Group 2 + 3. Regarding the BF component, Group 1 showed higher percentages of RTW-related problems in all categories as compared to Group 2 + 3, in particular for *b455 Exercise and tolerance functions* (85.7%), *b130 Energy and drive functions* (77.1%), and *b730 Muscle power functions* (60%), which were also the most relevant for Group 2 + 3 (b455 = 39.1%; b130 = 30.4%; b730 = 21.7%). Exceptions in this comparison were for three categories (*b1801 Body image*, *b830 Other functions of the skin*, and *b850 Functions of the air)*. As for the AP component, Group 1 showed higher percentages of RTW-related problems in all categories as compared to Group 2 + 3, except for two categories (*d166 Reading* and *d350 Conversation)*. Group 1 showed the highest percentages of RTW-related problems in the categories *d240 Handling stress and other psychological demands* (48.6%), *d415 Maintaining a body position* (42.9%), and *d430 Lifting and carrying objects* (40%), which were also the most relevant for Group 2 + 3 (d240 = 14.5%; d415 = 20.3%; d430 = 17.4%). Categories *d435 Moving objects with lower extremities* and *d510 Washing oneself* were not reported as problems by participants in either groups.

Supplementary Information 5 (SI5) presents the categories classified by the two groups of participants and the chapters of the EF component. Group 1 showed the highest percentages of factors influencing the RTW process in *e3 Support and relationship* (42.1%), *e1 Products and technology* (33.6%), and *e5 Health services, systems, and policies* (24.8%), which were also the most relevant for Group 2 + 3 (e3 = 23.5%; e1 = 13%; e5 = 8.7%). Group 1 showed the highest percentage of barriers in *e2 Natural environment and human-made changes to environment* (84.6%), *e4 Attitudes* (44.7%), and *e1 Products and technology* (40.4%); in addition to chapters e2 (90.9%) and e1 (47.2%), the most represented barriers in Group 2 + 3 were in the chapter *e5 Services, systems, and policies* (33.3%). Group 1 reported a higher proportion of barriers than did Group 2 + 3 only in the chapters *e3 Support and relationships* and *e4 Attitudes*.

Figure 2 shows the percentages of categories of the EF component reported by Group 1 and Group 2 + 3 participants, respectively. Group 1 showed higher percentages of barriers in all categories as compared to Group 2 + 3, except for four categories (*e240 Light*, *e250 Sound*, *e260 Air quality*, and *e325 Acquaintances, peers, colleagues, neighbors, and community members*). Group 1 showed the highest percentage of barriers in *e1101 Drugs* (42.9%), *e225 Climate* (25.7%), and *e330 People in position of authority* (20.0%), while Group 2 + 3 showed the highest percentage of barriers in *e1101 Drugs* (18.8%) and *e580 Health services, systems, and policies* (5.8%). Five categories (*e115 Products and technology for personal use in daily living, e320 Friends, e335 People in subordinate positions, e360* *Other professionals, e420 Individual attitudes of friends*) were not reported as barriers by participants in either group. Group 1 showed the highest percentage of facilitators in *e325 Acquaintances, peers, colleagues, neighbors and community members* (77.1%), *e310 Immediate family* (74.3%), *e320 Friends* (51.4%), and *e330 People in position of authority* (48.6%), which were also the most relevant for Group 2 + 3 (e325 = 50.7%; e310 = 43.5%; e330 = 40.6%; e320 = 24.6%).

### External validity

Concerning external validity, 40 (38.5%) participants had undergone chemotherapy (Group CT) and 64 (61.5%) had not (Group NoCT).

Supplementary Information 6 (SI6) describes the categories classified by treatment (CT) and the chapters of the BF and AP components. Concerning the BF component, Group CT showed higher percentages of RTW-related problems in all chapters than did Group NoCT, specifically in *b4 Exercise and tolerance functions* (48.8%), *b1 Mental functions* (27.8%), and *Other functions* (18.9%); these were also the most represented problems for Group NoCT, but with lower percentages (b1 = 21.4%; b4 = 18.8%; other functions = 8.9%). As for the AP component, Group CT showed higher percentages of problems in chapters *d2 General tasks and demands* (18.1%), *d4 Mobility* (14.5%), and *d8 Major life areas* (11.2%) as compared to Group NoCT. In addition to chapters d2 (15.2%) and d8 (11.0%), the third most represented problem in Group NoCT was in the chapter *e7 Interactions and interpersonal relationships* (9.7%).

**Fig. 3:**
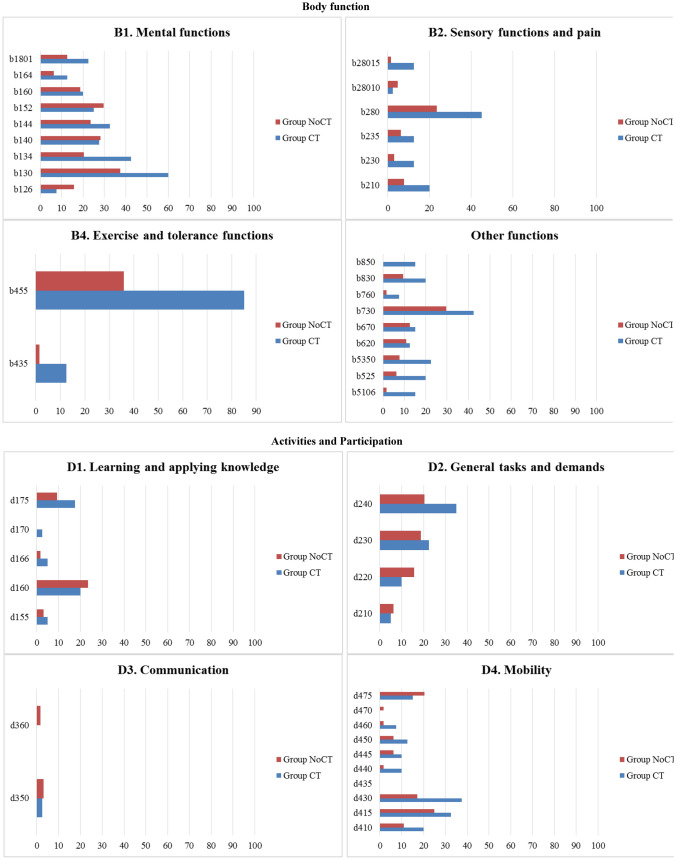

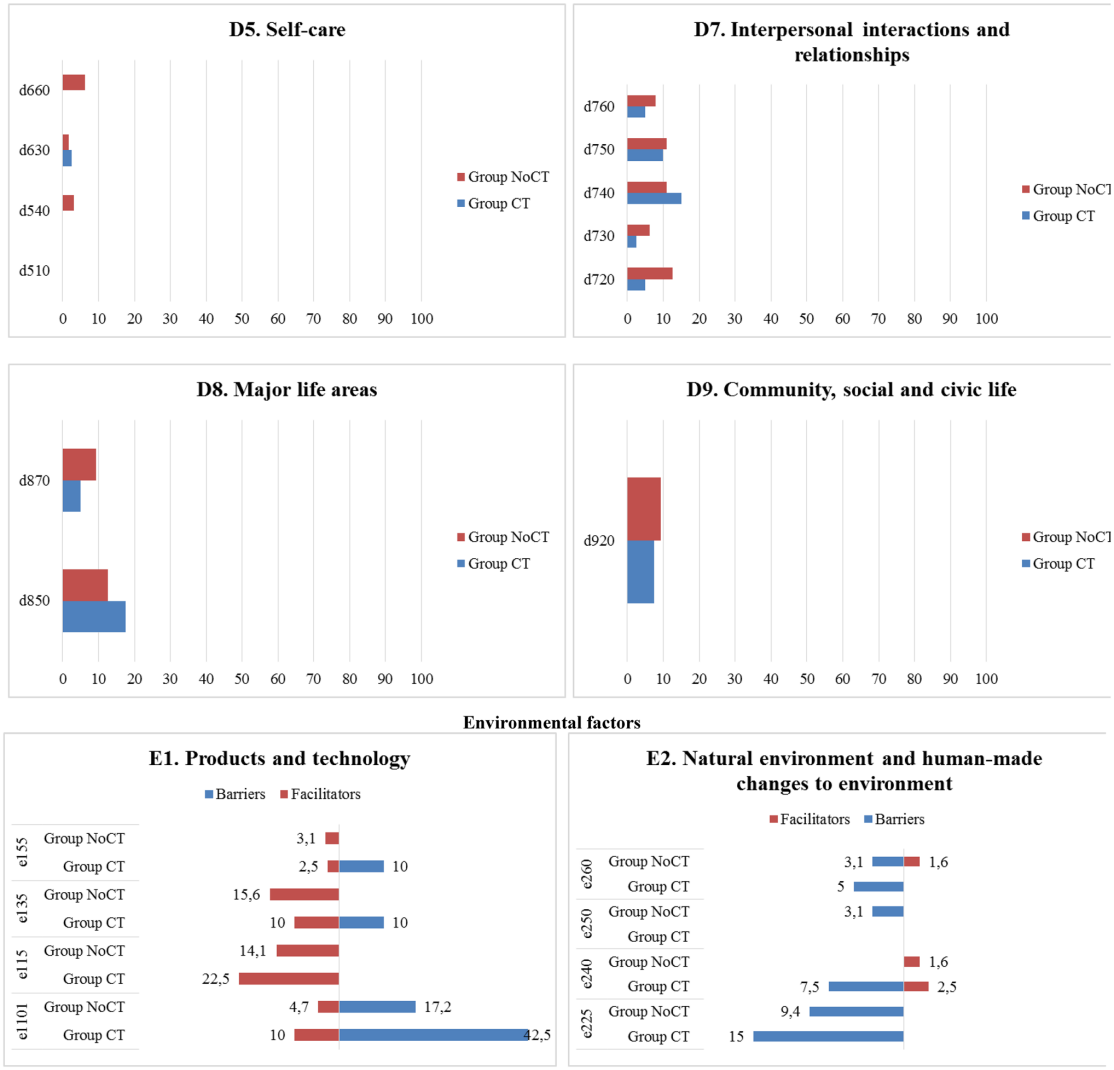

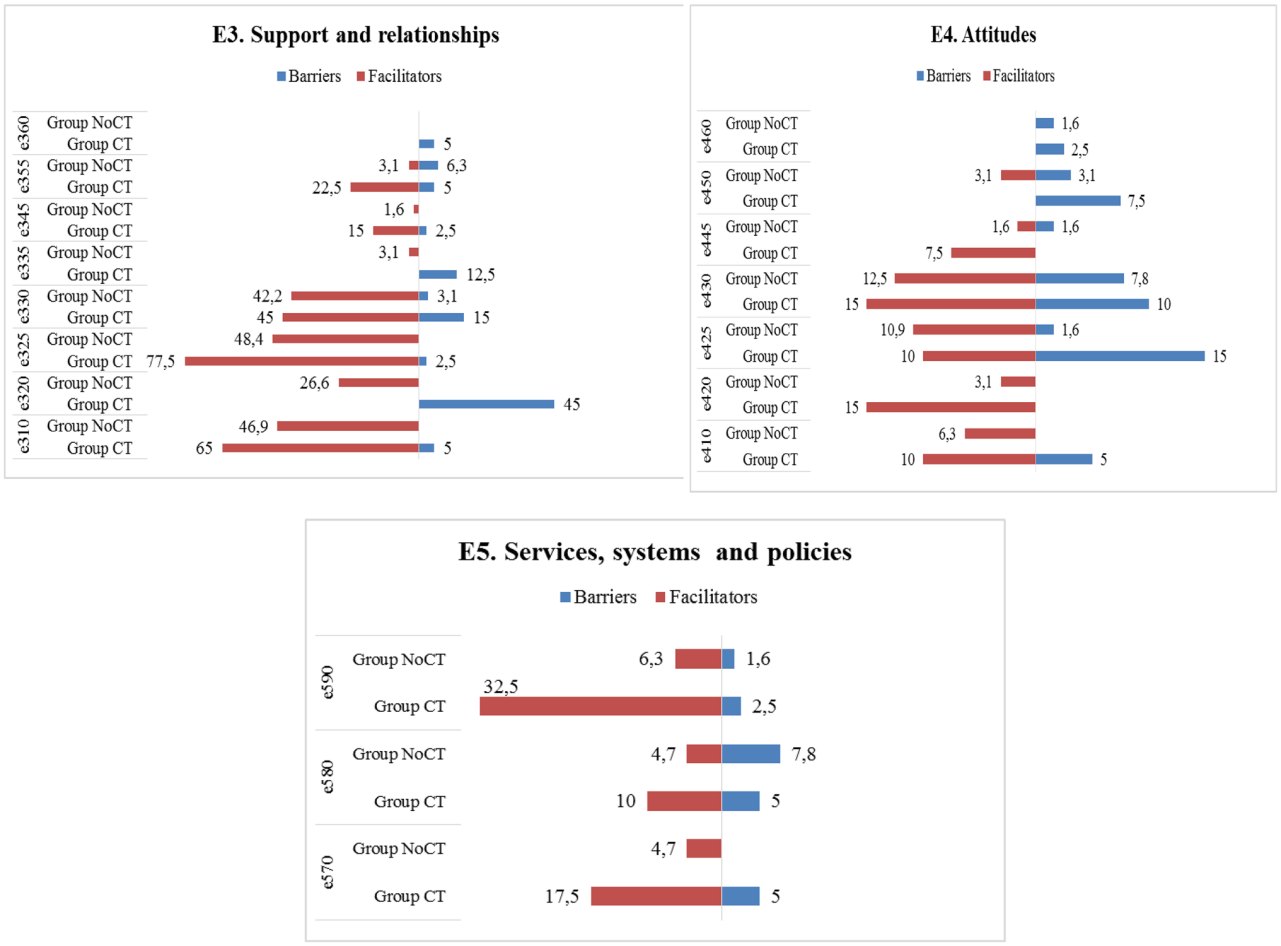
Description of categories **BF component.** b1. Mental functions: b126. Temperament and personality functions, b130. Energy and drive functions, b134. Sleep functions, b140. Attention functions, b144. Memory functions, b152. Emotional functions, b160. Thought functions, b164. Higher-level cognitive functions, b1801. Body image; b2. Sensory function and pain: b210. Seeing functions, b230. Hearing functions, b235. Vestibular functions, b280. Sensation of pain, b28010. Pain in head and neck, b28015. Pain in lower limb; b4. Exercise and tolerance functions: b435. Immunological system functions, b455. Exercise tolerance functions; Other functions: b5106. Functions of expelling the contents of the stomach, esophagus or pharynx, b525. Defecation functions, b5350. Sensation of nausea, b620. Urination functions, b670. Sensations associated with genital and reproductive functions, b730. Muscle power functions, b760. Control of voluntary movement functions, b830. Other functions of the skin, b850. Functions of hair. **AP component.** d1. Learning and applying knowledge: d155. Acquiring skills, d160. Focusing attention, d166. Reading, d170. Writing, d175. Solving problems; d2. General tasks and demands: d210. Undertaking a single task, d220. Undertaking multiple tasks, d230. Carrying out daily routine, d240. Handling stress and other psychological demands; d3. Communication: d350. Conversation, d360. Using communication devices and techniques; d4. Mobility: d410. Changing basic body position, d415. Maintaining a body position, d430. Lifting and carrying objects, d435. Moving objects with lower extremities, d440. Fine hand use, d445. Hand and arm use, d450. Walking, d460. Moving around in different locations, d470. Using transportation, d475. Driving; d5. Self-care and Domestic life: d510. Washing oneself, d540. Dressing, d630. Preparing meals, d660. Assisting others; d7. Interpersonal interactions and relationships: d720. Complex interpersonal interactions, d730. Relating with strangers, d740. Formal relationships, d750. Informal social relationships, d760. Family relationships; d8. Major life areas: d850. Remunerative employment, d870. Economic self-sufficiency; d9. Community, social and civic life: d920. Recreation and leisure. **EF component.** e1. Products and technology: e1101. Drugs, e115. Products and technology for personal use in daily living, e135. Products and technology for employment; e155. Design, construction and building products and technology of buildings for private use; e2. Natural environment and human-made changes to environment: e225. Climate, e240. Light, e250. Sound, e260. Air quality; e3. Support and relationships: e310. Immediate family, e320. Friends, e325. Acquaintances, peers, colleagues, neighbours and community members, e330. People in positions of authority, e335. People in subordinate positions, e345. Strangers, e355. Health professionals, e360 Other professionals; e4. Attitudes: e410. Individual attitudes of immediate family members, e420. Individual attitudes of friends, e425. Individual attitudes of acquaintances, peers, colleagues, neighbors, and community members, e430. Individual attitudes of people in positions of authority, e445. Individual attitudes of strangers, e450. Individual attitudes of health professionals, e460. Societal attitudes; e5. Services, systems, and policies: e570. Social security services, systems, and policies, e580. Health services, systems, and policies; e590. Labour and employment services, systems, and policies. Percentages of categories of the BF, AP, and EF components reported by participants who had and who had not undergone chemotherapy

Figure 3 shows the percentages of categories of the BF and AP components reported by participants in Group CT and in Group NoCT. As for the BF component, Group CT showed the highest percentages of problems in *b455 Exercise and tolerance functions* (85%), *b130 Energy and drive functions* (60%), and *b280 Sensation of pain* (45%) and higher percentages in all categories as compared to Group NoCT, except for four categories (*b126 Temperament and personality functions*, *b140 Attention functions*, *b152 Emotional functions*, and *b28010 Pain in head and neck)*. Regarding the AP component, Group CT showed higher percentages of problems in 16 categories as compared to Group NoCT, with the highest percentage in *d430 Lifting and carrying objects* (37.5%), *d240 Handling stress and other psychological demands* (35%), and *d415 Maintaining a body position* (32.5%); Group NoCT showed more problems in *d210 Undertaking a single task* (6.3%), *d220 Undertaking multiple tasks* (15.6%), *d160 Focusing attention* (23.4%), *d720 Complex interpersonal interactions* (12.5%), and *d475 Driving* (20.3%) as compared to Group CT, while *d430* was not a major issue (17.2%).

Supplementary Information 7 (SI7) describes the categories classified by treatment (CT) and the chapters of the EF component. Group CT reported more barriers in all chapters than did Group NoCT, except for *e5 Services, systems, and policies*, with the highest percentage of barriers in *e2 Natural environment and human-made changes to environment* (91.7%), *e1 Products and technology* (53.2%), and *e4 Attitudes* (38.1%). The pattern was similar for *e2 Natural environment and human-made changes to environment* (83.3%) and *e1 Products and technology* (30.6%) in the Group NoCT, but with lower percentages as compared to Group CT. As for the chapter *e3 Support and relationships*, ten cancer survivors in Group CT reported five or more categories that influenced their RTW process as compared to one cancer survivor in Group NoCT.

Figure 3 shows the percentages of categories of the EF component reported by participants in Group CT and in Group NoCT. Group CT showed higher percentages of barriers in all categories as compared to Group NoCT, except for four categories (*e250 Sound*, *e355 Health professionals*, *e445 Individuals attitudes of strangers, and e580 Health services, systems and policies*). Group CT showed the highest percentage of barriers in *e1101 Drugs* (42.5%), *e225 Climate* (15%), *e330 People in positions of authority* (15%), and *e425 Individual attitudes of acquaintances, peers, colleagues, neighbors, and community members* (15%). The pattern was similar for *e1101 Drugs* (17.2%) and *e225 Climate* (9.4%) in Group NoCT but with lower percentages. Group CT showed the highest percentage of facilitators in *e325 Acquaintances, peers, colleagues, neighbors and community members* (77.5%), *e310 Immediate family* (65%), *e330 People in position of authority* (45%), and *e320 Friends* (45%), which were also the most relevant for Group NoCT (e325 = 48.4%; e310 = 46.9%; e330 = 42.2%; e320 = 26.6%).

## Discussion

This cross-sectional study, the first step in validating the CS-VR-Onco and second phase of an exploratory mixed methods study, measured the tool’s validity in exploring cancer survivors’ perceived difficulties during the RTW process. The need to conduct studies with this aim has recently been highlighted due to the limited evidence supporting the use of the tool and to the growth of the cancer survivor population. Thus far, the CS-VR had been validated in only two populations, that of individuals with spinal cord injury, and that of healthcare professionals [22, 23].

The first phase of the exploratory mixed-methods study adopted qualitative methodology to adapt the original CS-VR to this population by using the lived experience of cancer survivors and stakeholders’ perspectives, resulting in the CS-VR-Onco [19]. In this second phase, we administered the CS-VR-Onco to a larger population of cancer survivors using a quantitative methodology to determine whether the tool was able to describe patients' problems and to detect any differences between the groups of participants. To the best of our knowledge, this is the first cross-sectional study aimed at validating a CS-VR for cancer survivors. In the absence of any gold standard of the constructs described by CS-VR-Onco for cancer survivors, two a priori hypotheses were formulated to reach a preliminary validation of this tool: 1) to assess internal validity, we chose to categorize the participants in groups based on their perceived RTW-related difficulties and the description of these in the components, chapters, and categories of the CS-VR-Onco; 2) to assess external validity, we chose to form two groups of participants based on the variable CT, i.e., whether or not they had undergone chemotherapy. The latter was formulated based on the results of previous studies [4, 24, 25]. From the distribution of the CS-VR-Onco categories, it was possible to describe the degree of validity of the tool and to report which categories were the most relevant in describing the differences between groups. The CS-VR-Onco was feasible as it was easy to administer, and all the questions were answered in a reasonable amount of time for both the patients and the interviewers. In our view, the tool is informative as it reflects the problems that a cancer survivor may have perceived during work reintegration. An additional strong point is that participants were able to answer all the questions.

The internal validity of the CS-VR-Onco was confirmed as the tool allows description of the RTW experiences of a large population of cancer survivors. Only seven categories out of 85 were never cited as problems or barriers, and participants did not report the lack of any relevant category to describe their RTW process. Moreover, the CS-VR-Onco detected differences between cancer survivors who reported having had RTW-related difficulties and those who reported no difficulties. Those reporting difficulties indicated more problems across all the components of the ICF than did those who reported not having had any RTW-related difficulty, indicating an overall impairment of their work functioning. In addition, the former may need a VR intervention, given the greater average number of problems reported (7.3 and 5.9 in BF and AP, respectively) as compared to participants who reported no difficulties (3.2 and 1.6 BF and AP, respectively). However, it is worth noting that both groups identified a high percentage of factors acting as facilitators, though higher in those reporting no difficulties. In almost all categories of the BF, AP, and EF (barriers) components, those reporting difficulties showed higher frequencies. Furthermore, when those reporting difficulties presented a high percentage of problems in one of the categories, it was much higher compared to those reporting no difficulties. For example, 77.1% of participants in the first group had problems in the category *b130 Energy and drive functions*, compared to 38.9% of participants in the second group. Instead, when those reporting difficulties reported a low percentage of problems, the differences between the two groups were minimal or the percentages of both groups were superimposable. We can hypothesize that those who reported difficulties also experienced problems in the less represented categories, but they may have placed less emphasis on these topics, and they may not have mentioned them during the interview because they may have had more possibly higher priority topics to report [27]. This could mean that patients who present many difficulties focus on the ones that have had the greatest impact on their experience of returning to work, leaving out some less important aspects.

The external validity of the CS-VR-Onco was confirmed as the participants who had undergone CT experienced more problems than those who had not undergone CT, as we hyphotesized based on results from previous studies [4, 24, 25]. Overall, our results contribute to gathering evidence to determine the external validity of this tool tool [28]. Although the observed trend was not applicable to four, fifteen, and four categories of the BF, AP and EF (barriers) components of the CS-VR-Onco, respectively, it is possible to explain the differences observed between Group CT and Group NoCT in light of the data from the literature. As is widely known, CT-related side effects impact many aspects of the BF component, for example, energy and fatigue [29], physical appearance [30], and pain [31]. Although the most affected functions in both groups were those relating to *b455 Exercise and tolerance functions*, *b130 Energy and drive functions*, and *b280 Sensations of pain*, the CT Group reported higher frequencies. Regarding the AP component, Group CT reported more problems in the categories *d430 Lifting and carrying objects* and *d240 Handling stress and other psychological demands*, which describe the ability to perform both psychologically and physically demanding work activities. An opposite trend was found for categories *d210 Undertaking single tasks* and *d220 Undertaking multiple tasks*, which were reported more frequently as problems by Group NoCT. A possible explanation of these differences concerns the fewer job accommodations in Group NoCT (34.8%) compared to Group CT (69.6%), in accordance with a previous study [32]. Regarding Chapter *d7 Interpersonal interactions and relationships*, Group NoCT seemed to have more problems. It is worth noting that Group NoCT also more frequently reported having had problems in the categories *b126 Temperament and personality functions* and *b152 Emotional functions* than did Group CT. Emotions and personality could have been more affected in participants who had not undergone CT because their relationship with healthcare professionals ended sooner [33] or because they perceived *e355 Health professionals* as barriers (6.3%) more than did Group CT (5%). Furthermore, cancer survivors who returned to work earlier may not have fully processed their emotions before returning to work [34, 35], encountering more obstacles in their relationships in the social work environment. Regarding the EF component, Group CT perceived barriers in each chapter more frequently than did Group NoCT, except for *e5 Services, systems and policies*. The outlier chapter can be explained by the fact that patients who undergo CT can usually obtain disability recognition during treatment and can use, for example, paid work permits [36] to make the RTW process more gradual. In terms of specific categories, both the CT and NoCT groups had high frequencies of barriers in *e1101 Drugs*, with Group CT having higher values. Finally, in terms of the social environment at work, while it is often facilitating, cancer survivors who had undergone CT had higher values of experiencing this environment as a barrier, particularly for the categories *e330 People in positions of authority* and *e425 Individual attitudes of acquaintances, peers, colleagues, neighbors, and community members*. Even though CT can lead to visible side effects and to prolonged health-related leaves of absence, relationships in the workplace can change [37], and discrimination can arise [38].

## Strengths and Limitations

The sample was randomly extracted from the Cancer Registry of the province of Reggio Emilia, an approach that led us to collet population-based data.

The main limitation of the study is that we could not validate the tool vs any gold standard. In this case, the absence of a gold standard is an ontological issue; the aim of the tool is not to assess the presence or absence of a condition but to map and describe the problems as well as analyze the barriers and facilitators. To overcome this problem, we proposed a concurrent validity approach. Nevertheless, we do not exclude that, at least for some specific topics, an applicable gold standard could be identified and that other concurrent validity measures could be used. Furthermore, although a tool that could help in the task of validating the CS-VR-Onco has been developed [17], it has not yet been culturally validated in Italian.

Because the CS-VR-Onco was adapted and preliminarily validated by recruiting cancer survivors who had returned to work, a possible limitation is that the tool may not include, or give low priority to, categories relevant to individuals who lost their job after their cancer diagnosis and who are looking for a new job. Furthermore, considering that the interviews took place about 3–4 years after diagnosis, there may have been recall bias in retrieving any RTW-related difficulty or CS-VR-Onco-described problem.

### Implications for Practice

The validation of the CS-VR-Onco will benefit the patient. Because the content of the CS-VR-Onco has proven to be relevant, this tool can describe all the problems that may occur during the RTW process, resulting in patients feeling that their work-related issues have not been neglected or overlooked. In addition, the CS-VR-Onco can also identify the barriers and facilitators associated with the environment, which patients often need to discuss as possible influencing factors. Furthermore, because the tool is relatively short and therefore quick to administer, it can be easier to use than the full ICF. Nevertheless, as the interviews lasted an average of 47 min, this amount of time may be too long in the clinical setting. It would be worth exploring whether to shorten the CS-VR-Onco by reducing the number of categories. One way this could be done may be by using the methodology employed by Finger et al. (2011) [14]: an international consensus conference was organized with stakeholders of VR who were called on to decide which categories to incude in the original CS-VR and in its short version. Thus, this approach could be used in future studies aiming at shortening the CS-VR-Onco. Furthermore, healthcare professionals could verify whether it is possible to first consider the categories that were more frequently reported in this study, such as *b455 Exercise and tolerance functions* and *e1101 Drugs*, as the assessment starting point. This hierarchical approach would make it possible to skip some chapters or categories if they are considered not applicable, thereby reducing the time of administration.

## Conclusions

The CS-VR-Onco proved to be feasible, with good response and applicability in a relatively large population of cancer survivors in a setting that was similar to its clinical application [39]. This tool was also able to identify more issues in cancer survivors who had experienced RTW-related difficulties and also identified differences between patients who had undergone CT, a known determinant of difficulties in RTW, and those who had not. These two results contribute to assessing the validity of this tool. In addition, our initial hypotheses were confirmed; the tool can identify the two characteristics of patients who may be candidate to VR: the perception of having had difficulties during the RTW process and having undergone CT. Furthermore, the results of this study suggest areas of the CS-VR-Onco whose content can be improved. The next step should be to study whether the CS-VR-Onco can appropriately identify the categories in which an intervention of VR is relevant and can identify the appropriate time of follow-up for each category of the CS-VR-Onco considered problematic. Finally, the most appropriate assessment tools to measure each category of the CS-VR-Onco have yet to be identified. In conclusion, the CS-VR-Onco can facilitate the description of the work functioning of the population of interest and can guide the multidisciplinary assessment of individuals who may require VR.

## Supplementary Information

Below is the link to the electronic supplementary material.

Supplementary file1 (DOCX 161 KB)

Supplementary file2 (DOCX 22 KB)

Supplementary file3 (DOC 63 KB)

Supplementary file4 (DOC 63 KB)

Supplementary file5 (DOC 113 KB)

Supplementary file6 (DOCX 31 KB)

Supplementary file7 (DOCX 45 KB)

## Data Availability

The datasets generated and/or analysed during the current study are available from the corresponding author on reasonable request.
