# Supplementary material for: Validating the Core Set for Vocational Rehabilitation in a Population of Cancer Survivors: A Cross-Sectional Study
Source: J Occup Rehabil. 2024 Dec 11;35(4):910–28. doi: 10.1007/s10926-024-10252-5 (PMC12575594; doi:10.1007/s10926-024-10252-5)
Supplement: Supplementary file 1 — Supplementary file1 (DOCX 161 KB) [file 10926_2024_10252_MOESM1_ESM.docx]

Date: ___/___/______

**Sociodemographic data**

| 1. **Sex** | □ male  □ female |
| --- | --- |
| 1. **Age** | ________ |
| 1. **Marital status** | □ married  □ single  □ widowed  □ divorced  □ separated  □ cohabitant |
| 1. **Education level** | □ no educational level  □ primary school  □ middle school  □ high school  □ university degree  □ other: _______________________________ |
| 1. **Children** | □ no  □ yes, how many? ____________ |

**Cancer-related factors**

| 1. **Did you undergo surgery?** | □ no  □ yes |
| --- | --- |
| 1. **Did you undergo surgery to remove the axillary lymph node or the adjacent lymph node?** | □ no  □ yes |
| 1. **Have you received radiotherapy?** | □ no  □ yes |
| 1. **Have you received chemotherapy?** | □ no  □ yes |
| 1. **How long did chemotherapy last?** | ……………………..months |
| 1. **Did you do hormone therapy?** | □ no  □ yes |
| 1. **Did you undergo bone marrow transplant?** | □ no  □ yes |

**Work-related factors**

**Before cancer diagnosis..**

| **13. What was your occupational status?** | □ employed  □ self-employed  □ other:  __________________________________________ |
| --- | --- |
| **14. What type of company did you work for?** | □ public sector  □ private sector  □ I do not know |
| **15. What was your job title and job description?** | _______________________________________________________________________________________________________________________________________________________________________________________________________________________ |
| **16. What type of contract did you have?** | □ permanent contract  □ fixed-term contract  □ other: ___________________________________________ |
| **17. What was your work schedule?** | □ full-time  □ part-time  □ other:  ___________________________________________ |
| **18. Where you a shift worker?** | □ no  □ yes |
| **19. Did you work in the evening? (from 8 pm to 11 pm)** | □ no  □ yes |
| **20. Did you work at night? (from 12 am to 5 am)** | □ no  □ yes |
| **21. Did you work on the weekend? (Saturdays and/or Sundays)** | □ no  □ yes |
| **22. Did you have flexible work schedule?** | □ no  □ yes |
| **23. Did you have flexible work tasks?** | □ no  □ yes |
| **24. How many people worked in your company?** | □ fewer than 10  □ from 10 to 49  □ from 50 to 249  □ > 249  □ alone |
| **25. How long did you have your job?** | □ under 1 year  □ from 1 to 4 years  □ from 5 to 10 years  □ more than 10 years |
| **26. Was your job psychologically demanding?** | □ no  □ yes  □ sometimes |
| **27. Was your job physically demanding?** | □ no  □ yes  □ sometimes |
| **28. When you returned to work, were any accommodations made?** | □ no  □ yes |
| **28bis.** **If so, which accommodations?** | □ more flexibility  □ work schedule reduction (i.e. from full-time to part-time)  □ change of work tasks  □ elimination of shifts (i.e. night shifts)  □ transferred to the workplace nearest home  □ other:  ___________________________________________ |
| **29. Did you encounter any difficulty during the return-to-work process or at work?** | □ no  □ yes |

**Which of the following categories were problems in your return to work or at work?**

**b126. Temperament and personality functions:** general mental functions of constitutional disposition of the individual to react in a particular way to situations, including the set of mental characteristics that makes the individual distinct from others.

| □ No, it wasn’t a problem | □ Yes, it was a problem |
| --- | --- |

**b126Modo.** How did it affect you?

_____________________________________________________________________________________________________________________________________________________________________________________________________________________________________________________________________

**b130. Energy and drive functions:** general mental functions of physiological and psychological mechanisms that cause the individual to move towards satisfying specific needs and general goals in a persistent manner.

| □ No, it wasn’t a problem | □ Yes, it was a problem |
| --- | --- |

**b130Modo.** How did it affect you?

_____________________________________________________________________________________________________________________________________________________________________________________________________________________________________________________________________

**b134. Sleep functions:** general mental functions of periodic, reversible, and selective physical and mental disengagement from one's immediate environment accompanied by characteristic physiological changes.

| □ No, it wasn’t a problem | □ Yes, it was a problem |
| --- | --- |

**b134Modo.** How did it affect you?

_____________________________________________________________________________________________________________________________________________________________________________________________________________________________________________________________________

**b140. Attention functions:** specific mental functions of focusing on an external stimulus or internal experience for the required period of time.

| □ No, it wasn’t a problem | □ Yes, it was a problem |
| --- | --- |

**b140Modo.** How did it affect you?

_____________________________________________________________________________________________________________________________________________________________________________________________________________________________________________________________________

**b144. Memory functions:** specific mental functions of registering and storing information and retrieving it as needed.

| □ No, it wasn’t a problem | □ Yes, it was a problem |
| --- | --- |

**b144Modo.** How did it affect you?

_____________________________________________________________________________________________________________________________________________________________________________________________________________________________________________________________________

**b152. Emotional functions:** specific mental functions related to the feeling and affective components of the processes of the mind.

| □ No, it wasn’t a problem | □ Yes, it was a problem |
| --- | --- |

**b152Modo.** How did it affect you?

_____________________________________________________________________________________________________________________________________________________________________________________________________________________________________________________________________

**b160. Thought functions:** specific mental functions related to the ideational component of the mind.

| □ No, it wasn’t a problem | □ Yes, it was a problem |
| --- | --- |

**b160Modo.** How did it affect you?

_____________________________________________________________________________________________________________________________________________________________________________________________________________________________________________________________________

**b164. Higher-level cognitive functions:** specific mental functions especially dependent on the frontal lobes of the brain, including complex goal-directed behaviours such as decision-making, abstract thinking, planning and carrying out plans, mental flexibility, and deciding which behaviours are appropriate under what circumstances; often called executive functions.

| □ No, it wasn’t a problem | □ Yes, it was a problem |
| --- | --- |

**b164Modo.** How did it affect you?

_____________________________________________________________________________________________________________________________________________________________________________________________________________________________________________________________________

**b1801. Body image:** specific mental functions related to the representation and awareness of one's body.

| □ No, it wasn’t a problem | □ Yes, it was a problem |
| --- | --- |

**b1801Modo.** How did it affect you?

_____________________________________________________________________________________________________________________________________________________________________________________________________________________________________________________________________

**b210.** **Seeing functions:** sensory functions relating to sensing the presence of light and sensing the form, size, shape and colour of the visual stimuli.

| □ No, it wasn’t a problem | □ Yes, it was a problem |
| --- | --- |

**b210Modo.** How did it affect you?

_____________________________________________________________________________________________________________________________________________________________________________________________________________________________________________________________________

**b230.** **Hearing functions:** sensory functions relating to sensing the presence of sounds and discriminating the location, pitch, loudness, and quality of sounds.

| □ No, it wasn’t a problem | □ Yes, it was a problem |
| --- | --- |

**b230Modo.** How did it affect you?

_____________________________________________________________________________________________________________________________________________________________________________________________________________________________________________________________________

**b235. Vestibular functions:** sensory functions of the inner ear related to position, balance, and movement.

| □ No, it wasn’t a problem | □ Yes, it was a problem |
| --- | --- |
|  |  |

**b235Modo.** How did it affect you?

_____________________________________________________________________________________________________________________________________________________________________________________________________________________________________________________________________

**b280. Sensation of pain:** sensation of unpleasant feeling indicating potential or actual damage to some body structure.

| □ No, it wasn’t a problem | □ Yes, it was a problem |
| --- | --- |

**b280Modo.** How did it affect you?

_____________________________________________________________________________________________________________________________________________________________________________________________________________________________________________________________________

**b28010. Pain in head and neck:** sensation of unpleasant feeling indicating potential or actual damage to some body structure felt in the head and neck.

| □ No, it wasn’t a problem | □ Yes, it was a problem |
| --- | --- |

**b28010Modo.** How did it affect you?

_____________________________________________________________________________________________________________________________________________________________________________________________________________________________________________________________________

**b28015. Pain in lower limb:** sensation of unpleasant feeling indicating potential or actual damage to some body structure felt in either one or both lower limbs, including feet.

| □ No, it wasn’t a problem | □ Yes, it was a problem |
| --- | --- |

**b28015Modo.** How did it affect you?

_____________________________________________________________________________________________________________________________________________________________________________________________________________________________________________________________________

**b435. Immunological system functions:** functions of the body related to protection against foreign substances, including infections, by specific and non-specific immune responses.

| □ No, it wasn’t a problem | □ Yes, it was a problem |
| --- | --- |

**b435Modo.** How did it affect you?

_____________________________________________________________________________________________________________________________________________________________________________________________________________________________________________________________________

**b455. Exercise tolerance functions:** functions related to respiratory and cardiovascular capacity as required for enduring physical exertion.

| □ No, it wasn’t a problem | □ Yes, it was a problem |
| --- | --- |

**b455Modo.** How did it influence you?

_____________________________________________________________________________________________________________________________________________________________________________________________________________________________________________________________________

**b5106. Functions of expelling the contents of the stomach, oesophagus or pharynx:** functions of moving substances such as food, liquid, medications, objects intentionally or unintentionally swallowed in the reverse direction to ingestion, such as in regurgitation and vomiting.

| □ No, it wasn’t a problem | □ Yes, it was a problem |
| --- | --- |

**b5106Modo.** How did it affect you?

_____________________________________________________________________________________________________________________________________________________________________________________________________________________________________________________________________

**b525. Defecation functions:** functions of elimination of wastes and undigested food as faeces and related functions.

| □ No, it wasn’t a problem | □ Yes, it was a problem |
| --- | --- |

**b525Modo.** How did it affect you?

_____________________________________________________________________________________________________________________________________________________________________________________________________________________________________________________________________

**b5350. Sensation of nausea:** sensation of needing to vomit.

| □ No, it wasn’t a problem | □ Yes, it was a problem |
| --- | --- |

**b5350Modo.** How did it affect you?

_____________________________________________________________________________________________________________________________________________________________________________________________________________________________________________________________________

**b620. Urination functions:** functions of discharge of urine from the urinary bladder.

| □ No, it wasn’t a problem | □ Yes, it was a problem |
| --- | --- |

**b620Modo.** How did it affect you?

_____________________________________________________________________________________________________________________________________________________________________________________________________________________________________________________________________

**b670. Sensations associated with genital and reproductive functions:** sensations associated with sexual intercourse, menstruation, and related genital or reproductive functions

| □ No, it wasn’t a problem | □ Yes, it was a problem |
| --- | --- |

**b670Modo.** How did it affect you?

_____________________________________________________________________________________________________________________________________________________________________________________________________________________________________________________________________

**b730. Muscle power functions:** functions related to the force generated by the contraction of a muscle or muscle groups.

| □ No, it wasn’t a problem | □ Yes, it was a problem |
| --- | --- |

**b730Modo.** How did it affect you?

_____________________________________________________________________________________________________________________________________________________________________________________________________________________________________________________________________

**b760. Control of voluntary movement functions:** functions associated with control over and coordination of voluntary movements.

| □ No, it wasn’t a problem | □ Yes, it was a problem |
| --- | --- |

**b760Modo.** How did it affect you?

_____________________________________________________________________________________________________________________________________________________________________________________________________________________________________________________________________

**b830. Other functions of the skin:** functions of the skin other than protection and repair, such as cooling and sweat secretion.

| □ No, it wasn’t a problem | □ Yes, it was a problem |
| --- | --- |

**b830Modo.** How did it affect you?

_____________________________________________________________________________________________________________________________________________________________________________________________________________________________________________________________________

**b850. Functions of hair:** functions of the hair, such as protection, coloration and appearance.

| □ No, it wasn’t a problem | □ Yes, it was a problem |
| --- | --- |

**b850Modo.** How did it affect you?

_____________________________________________________________________________________________________________________________________________________________________________________________________________________________________________________________________

**d155. Acquiring skills:** developing basic and complex competencies in integrated sets of actions or tasks so as to initiate and follow through with the acquisition of a skill, such as manipulating tools or toys or playing games.

| □ No, it wasn’t a problem | □ Yes, it was a problem |
| --- | --- |

**d155Modo.** How did it affect you?

_____________________________________________________________________________________________________________________________________________________________________________________________________________________________________________________________________

**d160.  Focusing attention:** intentionally focusing on specific stimuli, such as by filtering out distracting noises.

| □ No, it wasn’t a problem | □ Yes, it was a problem |
| --- | --- |

**d160Modo.** How did it affect you?

_____________________________________________________________________________________________________________________________________________________________________________________________________________________________________________________________________

**d166. Reading:** performing activities involved in the comprehension and interpretation of written language (e.g. books, instructions or newspapers in text or Braille), for the purpose of obtaining general knowledge or specific information.

| □ No, it wasn’t a problem | □ Yes, it was a problem |
| --- | --- |

**d166Modo.** How did it affect you?

_____________________________________________________________________________________________________________________________________________________________________________________________________________________________________________________________________

**d170. Writing:** using or producing symbols or language to convey information, such as producing a written record of events or ideas or drafting a letter.

| □ No, it wasn’t a problem | □ Yes, it was a problem |
| --- | --- |

**d170Modo.** How did it affect you?

_____________________________________________________________________________________________________________________________________________________________________________________________________________________________________________________________________

**d175. Solving problems:** finding solutions to questions or situations by identifying and analysing issues, developing options and solutions, evaluating potential effects of solutions, and executing a chosen solution, such as in resolving a dispute between two people.

| □ No, it wasn’t a problem | □ Yes, it was a problem |
| --- | --- |

**d175Modo.** How did it affect you?

_____________________________________________________________________________________________________________________________________________________________________________________________________________________________________________________________________

**d210. Undertaking a single task:** carrying out simple or complex and coordinated actions related to the mental and physical components of a single task, such as initiating a task, organizing time, space and materials for a task, pacing task performance, and carrying out, completing, and sustaining a task.

| □ No, it wasn’t a problem | □ Yes, it was a problem |
| --- | --- |

**d210Modo.** How did it affect you?

_____________________________________________________________________________________________________________________________________________________________________________________________________________________________________________________________________

**d220. Undertaking multiple tasks:** carrying out simple or complex and coordinated actions as components of multiple, integrated and complex tasks in sequence or simultaneously.

| □ No, it wasn’t a problem | □ Yes, it was a problem |
| --- | --- |

**d220Modo.** How did it affect you?

_____________________________________________________________________________________________________________________________________________________________________________________________________________________________________________________________________

**d230. Carrying out daily routine:** carrying out simple or complex and coordinated actions in order to plan, manage, and complete the requirements of day-to-day procedures or duties, such as budgeting time and making plans for separate activities throughout the day.

| □ No, it wasn’t a problem | □ Yes, it was a problem |
| --- | --- |

**d230Modo.** How did it affect you?

_____________________________________________________________________________________________________________________________________________________________________________________________________________________________________________________________________

**d240. Handling stress and other psychological demands:** carrying out simple or complex and coordinated actions to manage and control the psychological demands required to carry out tasks demanding significant responsibilities and involving stress, distraction, or crises, such as driving a vehicle during heavy traffic or taking care of many children.

| □ No, it wasn’t a problem | □ Yes, it was a problem |
| --- | --- |

**d240Modo.** How did it affect you?

_____________________________________________________________________________________________________________________________________________________________________________________________________________________________________________________________________

**d350. Conversation:** starting, sustaining, and ending an interchange of thoughts and ideas carried out by means of spoken, written, signed, or other forms of language with one or more people one knows or who are strangers, in formal or casual settings.

| □ No, it wasn’t a problem | □ Yes, it was a problem |
| --- | --- |

**d350Modo.** How did it affect you?

_____________________________________________________________________________________________________________________________________________________________________________________________________________________________________________________________________

**d360. Using communication devices and techniques:** using devices, techniques, and other means for the purposes of communicating, such as calling a friend on the telephone.

| □ No, it wasn’t a problem | □ Yes, it was a problem |
| --- | --- |

**d360Modo.** How did it affect you?

_____________________________________________________________________________________________________________________________________________________________________________________________________________________________________________________________________

**d410. Changing basic body position:** getting into and out of a body position and moving from one location to another, such as getting up out of a chair to lie down on a bed, and getting into and out of positions of sitting, standing, kneeling, or squatting.

| □ No, it wasn’t a problem | □ Yes, it was a problem |
| --- | --- |

**d410Modo.** How did it affect you?

_____________________________________________________________________________________________________________________________________________________________________________________________________________________________________________________________________

**d415. Maintaining a body position:** staying in the same body position as required, such as remaining seated or remaining standing for to carrying out a task, in play, work, or school.

| □ No, it wasn’t a problem | □ Yes, it was a problem |
| --- | --- |

**d415Modo.** How did it affect you?

_____________________________________________________________________________________________________________________________________________________________________________________________________________________________________________________________________

**d430. Lifting and carrying objects:** raising up an object or taking something from one place to another, such as when lifting a cup or toy or carrying a box, or a child from one room to another.

| □ No, it wasn’t a problem | □ Yes, it was a problem |
| --- | --- |

**d430Modo.** How did it affect you?

_____________________________________________________________________________________________________________________________________________________________________________________________________________________________________________________________________

**d435. Moving objects with lower extremities:** performing coordinated actions aimed at moving an object by using the legs and feet, such as kicking a ball or pushing pedals on a bicycle.

| □ No, it wasn’t a problem | □ Yes, it was a problem |
| --- | --- |

**d435Modo.** How did it affect you?

_____________________________________________________________________________________________________________________________________________________________________________________________________________________________________________________________________

**d440. Fine hand use:** performing the coordinated actions of handling objects, picking up, manipulating and releasing them using one's hand, fingers, and thumb, such as required to lift coins off a table or turn a dial or knob.

| □ No, it wasn’t a problem | □ Yes, it was a problem |
| --- | --- |

**d440Modo.** How did it affect you?

_____________________________________________________________________________________________________________________________________________________________________________________________________________________________________________________________________

**d445. Hand and arm use:** performing the coordinated actions required to move objects or to manipulate them by using hands and arms, such as when turning door handles or throwing or catching an object.

| □ No, it wasn’t a problem | □ Yes, it was a problem |
| --- | --- |

**d445Modo.** How did it affect you?

_____________________________________________________________________________________________________________________________________________________________________________________________________________________________________________________________________

**d450. Walking:** moving along a surface on foot, step by step, so that one foot is always on the ground, such as when strolling, sauntering, walking forwards, backwards, or sideways.

| □ No, it wasn’t a problem | □ Yes, it was a problem |
| --- | --- |

**d450Modo.** How did it affect you?

_____________________________________________________________________________________________________________________________________________________________________________________________________________________________________________________________________

**d460. Moving around in different locations:** walking and moving around in various places and situations, such as walking between rooms in a house, within a building, or down the street of a town.

| □ No, it wasn’t a problem | □ Yes, it was a problem |
| --- | --- |

**d460Modo.** How did it affect you?

_____________________________________________________________________________________________________________________________________________________________________________________________________________________________________________________________________

**d470. Using transportation**: using transportation to move around as a passenger, such as being driven in a car, bus, rickshaw, jitney, pram or stroller, wheelchair, animal-powered vehicle, private or public taxi, train, tram, subway, boat or aircraft and using humans for transportation.

| □ No, it wasn’t a problem | □ Yes, it was a problem |
| --- | --- |

**d470Modo.** How did it affect you?

_____________________________________________________________________________________________________________________________________________________________________________________________________________________________________________________________________

**d475. Driving:** being in control of and moving a vehicle or the animal that draws it, travelling under one's own direction or having at one's disposal any form of transportation appropriate for age, such as a car, bicycle, boat or animal powered vehicles.

| □ No, it wasn’t a problem | □ Yes, it was a problem |
| --- | --- |

**d475Modo**. How did it affect you?

_____________________________________________________________________________________________________________________________________________________________________________________________________________________________________________________________________

**d510. Washing oneself:** washing and drying one's whole body, or body parts, using water and appropriate cleaning and drying materials or methods, such as bathing, showering, washing hands and feet, face and hair, and drying with a towel.

| □ No, it wasn’t a problem | □ Yes, it was a problem |
| --- | --- |

**d510Modo**. How did it affect you?

_____________________________________________________________________________________________________________________________________________________________________________________________________________________________________________________________________

**d540. Dressing:** carrying out the coordinated actions and tasks of putting on and taking off clothes and footwear in sequence and in keeping with climatic and social conditions, such as by putting on, adjusting and removing shirts, skirts, blouses, pants, undergarments, saris, kimono, tights, hats, gloves, coats, shoes, boots, sandals and slippers.

| □ No, it wasn’t a problem | □ Yes, it was a problem |
| --- | --- |

**d540Modo**. How did it affect you?

_____________________________________________________________________________________________________________________________________________________________________________________________________________________________________________________________________

**d630. Preparing meals:** planning, organizing, cooking, and serving simple and complex meals for oneself and others, such as by making a menu, selecting edible food and drink, getting together ingredients for preparing meals, cooking with heat and preparing cold foods and drinks, and serving the food.

| □ No, it wasn’t a problem | □ Yes, it was a problem |
| --- | --- |

**d630Modo**. How did it affect you?

_____________________________________________________________________________________________________________________________________________________________________________________________________________________________________________________________________

**d660. Assisting others:** assisting household members and others with their learning, communicating, self-care, movement, within the house or outside; being concerned about the well-being of household members and others.

| □ No, it wasn’t a problem | □ Yes, it was a problem |
| --- | --- |

**d660Modo**. How did it affect you?

_____________________________________________________________________________________________________________________________________________________________________________________________________________________________________________________________________

**d720. Complex interpersonal interactions:** maintaining and managing interactions with other people, in a contextually and socially appropriate manner, such as by regulating emotions and impulses, controlling verbal and physical aggression, acting independently in social interactions, and acting in accordance with social rules and conventions, when for example playing, studying or working with others.

| □ No, it wasn’t a problem | □ Yes, it was a problem |
| --- | --- |

**d720Modo**. How did it affect you?

_____________________________________________________________________________________________________________________________________________________________________________________________________________________________________________________________________

**d730. Relating with strangers:** engaging in temporary contacts and links with strangers for specific purposes, when asking for directions or other information, or making a purchase.

| □ No, it wasn’t a problem | □ Yes, it was a problem |
| --- | --- |

**d730Modo**. How did it affect you?

_____________________________________________________________________________________________________________________________________________________________________________________________________________________________________________________________________

**d740. Formal relationships:** creating and maintaining specific relationships in formal settings, such as with teachers, employers, professionals, or service providers.

| □ No, it wasn’t a problem | □ Yes, it was a problem |
| --- | --- |

**d7405Modo**. How did it affect you?

_____________________________________________________________________________________________________________________________________________________________________________________________________________________________________________________________________

**d750. Informal social relationships:** entering into relationships with others, such as casual relationships with people living in the same community or residence, or with co-workers, students, playmates, people with similar backgrounds or professions.

| □ No, it wasn’t a problem | □ Yes, it was a problem |
| --- | --- |

**d750Modo**. How did it affect you?

_____________________________________________________________________________________________________________________________________________________________________________________________________________________________________________________________________

**d760. Family relationships:** creating and maintaining kinship relationships, such as those with members of the nuclear family, extended family, foster and adopted family and step-relationships, more distant relationships such as second cousins, or legal guardians

| □ No, it wasn’t a problem | □ Yes, it was a problem |
| --- | --- |

**d760Modo**. How did it affect you?

_____________________________________________________________________________________________________________________________________________________________________________________________________________________________________________________________________

**d850. Remunerative employment:** engaging in all aspects of work, as an occupation, trade, profession or other form of employment, for payment, as an employee, full or part time, or self-employed, such as seeking employment and getting a job, doing the required tasks of the job, attending work on time as required, supervising other workers or being supervised, and performing required tasks alone or in groups.

| □ No, it wasn’t a problem | □ Yes, it was a problem |
| --- | --- |

**d850Modo**. How did it affect you?

_____________________________________________________________________________________________________________________________________________________________________________________________________________________________________________________________________

**d870. Economic self-sufficiency:** having command over economic resources, from private or public sources, in order to ensure economical security for present and future needs.

| □ No, it wasn’t a problem | □ Yes, it was a problem |
| --- | --- |

**d870Modo**. How did it affect you?

_____________________________________________________________________________________________________________________________________________________________________________________________________________________________________________________________________

**d920. Recreation and leisure:** engaging in any form of play, recreational or leisure activity, such as informal or organized play and sports, programmes of physical fitness, relaxation, amusement or diversion, going to art galleries, museums, cinemas or theatres; engaging in crafts or hobbies, reading for enjoyment, playing musical instruments; sightseeing, tourism and travelling for pleasure.

| □ No, it wasn’t a problem | □ Yes, it was a problem |
| --- | --- |

**d920Modo**. How did it affect you?

_____________________________________________________________________________________________________________________________________________________________________________________________________________________________________________________________________

**Which of the following categories influenced your return to work or ability to work?**

**Of the following categories, which have been barriers or facilitators in your return to work or continuation of work?**

**e1101. Drugs:** any natural or human-made object or substance gathered, processed or manufactured for medicinal purposes, such as allopathic and naturopathic medication.

| □ No, it didn’t | □ Yes, it did |
| --- | --- |

**e1101bis.** How did it affect you?

| □ Barrier | □ Facilitator |
| --- | --- |

**e110tris.** Why do you think this category was a barrier or facilitator?

_____________________________________________________________________________________________________________________________________________________________________________________________________________________________________________________________________

**e115. Products and technology for personal use in daily living:** equipment, products, and technologies used by people in daily activities, including those adapted or specially designed, located in, on, or near the person using them

| □ No, it didn’t | □ Yes, it did |
| --- | --- |

**e115bis.** How did it affect you?

| □ Barrier | □ Facilitator |
| --- | --- |
|  |  |

**e115tris.** Why do you think this category was a barrier or facilitator?

_____________________________________________________________________________________________________________________________________________________________________________________________________________________________________________________________________

**e135. Products and technology for employment:** equipment, products, and technology used for employment to facilitate work activities.

| □ No, it didn’t | □ Yes, it did |
| --- | --- |

**e135bis.** How did it affect you?

| □ Barrier | □ Facilitator |
| --- | --- |

**e135tris.** Why do you think this category was a barrier or facilitator?

**e155. Design, construction and building products and technology of buildings for private use:** product and technology that constitute an individual's indoor and outdoor human-made environment that is planned, designed and constructed for private use (e.g. home, dwelling), including those adapted or specially designed.

| □ No, it didn’t | □ Yes, it did |
| --- | --- |

**e155bis.** How did it affect you?

| □ Barrier | □ Facilitator |
| --- | --- |

**e155tris.** Why do you think this category was a barrier or facilitator?

_____________________________________________________________________________________________________________________________________________________________________________________________________________________________________________________________________

**e225. Climate:** meteorological features and events, such as the weather.

| □ No, it didn’t | □ Yes, it did |
| --- | --- |

**e225bis.** How did it affect you?

| □ Barrier | □ Facilitator |
| --- | --- |

**e225tris.** Why do you think this category was a barrier or facilitator?

_____________________________________________________________________________________________________________________________________________________________________________________________________________________________________________________________________

**e240. Light:** electromagnetic radiation by which things are made visible by either sunlight or artificial lighting (e.g. candles, oil paraffin lamps and electricity), and which may provide useful or distracting information about the world.

| □ No, it didn’t | □ Yes, it did |
| --- | --- |

**e240bis.** How did it affect you?

| □ Barrier | □ Facilitator |
| --- | --- |

**e240tris.** Why do you think this category was a barrier or facilitator?

______________________________________________________________________________________________________________________________________________________________________________

**e250. Sound:** a phenomenon that is or may be heard, such as banging, ringing, thumping, whistling, yelling or buzzing, in any volume, timbre or tone, and that may provide useful or distracting information about the world.

| □ No, it didn’t | □ Yes, it did |
| --- | --- |

**e250bis.** How did it affect you?

| □ Barrier | □ Facilitator |
| --- | --- |

**e250tris.** Why do you think this category was a barrier or facilitator?

_____________________________________________________________________________________________________________________________________________________________________________________________________________________________________________________________________

**e260. Air quality:** characteristics of the atmosphere (outside buildings) or enclosed areas of air (inside buildings), and which may provide useful or distracting information about the world.

| □ No, it didn’t | □ Yes, it did |
| --- | --- |

**e260bis.** How did it affect you?

| □ Barrier | □ Facilitator |
| --- | --- |

**e260tris.** Why do you think this category was a barrier or facilitator?

_____________________________________________________________________________________________________________________________________________________________________________________________________________________________________________________________________

**e310. Immediate family:** individuals related by birth, marriage or other relationship recognized by the culture as immediate family, such as spouses, partners, parents, siblings, children, foster parents, adoptive parents and grandparents.

| □ No, it didn’t | □ Yes, it did |
| --- | --- |

**e310bis.** How did it affect you?

| □ Barrier | □ Facilitator |
| --- | --- |

**e310tris.** Why do you think this category was a barrier or facilitator?

_____________________________________________________________________________________________________________________________________________________________________________________________________________________________________________________________________

**e320. Friends:** individuals who are close and ongoing participants in relationships characterized by trust and mutual support.

| □ No, it didn’t | □ Yes, it did |
| --- | --- |

**e320bis.** How did it affect you?

| □ Barrier | □ Facilitator |
| --- | --- |

**e320tris.** Why do you think this category was a barrier or facilitator?

_____________________________________________________________________________________________________________________________________________________________________________________________________________________________________________________________________

**e325. Acquaintances, peers, colleagues, neighbours and community members:** individuals who are familiar to each other as acquaintances, peers, colleagues, neighbours, and community members, in situations of work, school, recreation, or other aspects of life, and who share demographic features such as age, gender, religious creed or ethnicity or pursue common interests.

| □ No, it didn’t | □ Yes, it did |
| --- | --- |

**e325bis.** How did it affect you?

| □ Barrier | □ Facilitator |
| --- | --- |

**e325tris.** Why do you think this category was a barrier or facilitator?

_____________________________________________________________________________________________________________________________________________________________________________________________________________________________________________________________________

**e330. People in positions of authority:** individuals who have decision-making responsibilities for others and who have socially defined influence or power based on their social, economic, cultural or religious roles in society, such as teachers, employers, supervisors, religious leaders, substitute decision-makers, guardians or trustees.

| □ No, it didn’t | □ Yes, it did |
| --- | --- |

**e330bis.** How did it affect you?

| □ Barrier | □ Facilitator |
| --- | --- |

**e330tris.** Why do you think this category was a barrier or facilitator?

**e335. People in subordinate positions:** individuals whose day-to-day life is influenced by people in positions of authority in work, school or other settings, such as students, workers, and members of a religious group.

| □ No, it didn’t | □ Yes, it did |
| --- | --- |

**e335bis.** How did it affect you?

| □ Barrier | □ Facilitator |
| --- | --- |

**e335tris.** Why do you think this category was a barrier or facilitator?

_____________________________________________________________________________________________________________________________________________________________________________________________________________________________________________________________________

**e345. Strangers:** individuals who are unfamiliar and unrelated, or those who have not yet established a relationship or association, including persons unknown to the individual but who are sharing a life situation with them, such as substitute teachers, co-workers or care providers.

| □ No, it didn’t | □ Yes, it did |
| --- | --- |

**e345bis.** How did it affect you?

| □ Barrier | □ Facilitator |
| --- | --- |

**e345tris.** Why do you think this category was a barrier or facilitator?

_____________________________________________________________________________________________________________________________________________________________________________________________________________________________________________________________________

**e355. Health professionals:** all service providers working within the context of the health system, such as doctors, nurses, physiotherapists, occupational therapists, speech therapists, audiologists, orthotist-prosthetists, medical social workers.

| □ No, it didn’t | □ Yes, it did |
| --- | --- |

**e355bis.** How did it affect you?

| □ Barrier | □ Facilitator |
| --- | --- |

**e355tris.** Why do you think this category was a barrier or facilitator?

_____________________________________________________________________________________________________________________________________________________________________________________________________________________________________________________________________

**e360. Other professionals**: all service providers working outside of the health system, including social workers, lawyers, teachers, architects, and designers.

| □ No, it didn’t | □ Yes, it did |
| --- | --- |

**e360bis.** How did it affect you?

| □ Barrier | □ Facilitator |
| --- | --- |

**e360tris.** Why do you think this category was a barrier or facilitator?

_____________________________________________________________________________________________________________________________________________________________________________________________________________________________________________________________________

**e410. Individual attitudes of immediate family members:** general or specific opinions and beliefs of immediate family members about the person or about other matters (e.g. social, political and economic issues), that influence individual behaviour and actions.

| □ No, it didn’t | □ Yes, it did |
| --- | --- |

**e410bis.** How did it affect you?

| □ Barrier | □ Facilitator |
| --- | --- |

**e410tris.** Why do you think this category was a barrier or facilitator?

_____________________________________________________________________________________________________________________________________________________________________________________________________________________________________________________________________

**e420. Individual attitudes of friends:** general or specific opinions and beliefs of friends about the person or about other matters, (e.g. social, political and economic issues), that influence individual behaviour and actions

| □ No, it didn’t | □ Yes, it did |
| --- | --- |

**e420bis.** How did it affect you?

| □ Barrier | □ Facilitator |
| --- | --- |

**e420tris.** Why do you think this category was a barrier or facilitator?

_____________________________________________________________________________________________________________________________________________________________________________________________________________________________________________________________________

**e425. Individual attitudes of acquaintances, peers, colleagues, neighbours and community members:** general or specific opinions and beliefs of acquaintances, peers, colleagues, neighbours, and community members about the person or about other matters, (e.g. social, political and economic issues), that influence individual behaviour and actions.

| □ No, it didn’t | □ Yes, it did |
| --- | --- |

**e425bis.** How did it affect you?

| □ Barrier | □ Facilitator |
| --- | --- |

**e425tris.** Why do you think this category was a barrier or facilitator?

_____________________________________________________________________________________________________________________________________________________________________________________________________________________________________________________________________

**e430. Individual attitudes of people in positions of authority:** general or specific opinions and beliefs of people in positions of authority about the person or about other matters, (e.g. social, political and economic issues), that influence individual behaviour and actions.

| □ No, it didn’t | □ Yes, it did |
| --- | --- |

**e430bis.** How did it influence you?

| □ Barrier | □ Facilitator |
| --- | --- |

**e430tris.** Why do you think this category was a barrier or facilitator?

_____________________________________________________________________________________________________________________________________________________________________________________________________________________________________________________________________

**e445. Individual attitudes of strangers:** general or specific opinions and beliefs of strangers about the person or about other matters, (e.g. social, political and economic issues), that influence individual behaviour and actions.

| □ No, it didn’t | □ Yes, it did |
| --- | --- |

**e445bis.** How did it influence you?

| □ Barrier | □ Facilitator |
| --- | --- |

**e445tris.** Why do you think this category was a barrier or facilitator?

_____________________________________________________________________________________________________________________________________________________________________________________________________________________________________________________________________

**e450. Individual attitudes of health professionals:** general or specific opinions and beliefs of health professionals about the person or about other matters, (e.g. social, political and economic issues), that influence individual behaviour and actions.

| □ No, it didn’t | □ Yes, it did |
| --- | --- |

**e450bis.** How did it influence you?

| □ Barrier | □ Facilitator |
| --- | --- |

**e450tris.** Why do you think this category was a barrier or facilitator?

_____________________________________________________________________________________________________________________________________________________________________________________________________________________________________________________________________

**e460. Societal attitudes:** general or specific opinions and beliefs generally held by people of a culture, society, sub-cultural or other social group about other individuals or about other social, political and economic issues, that influence group or individual behaviour and actions.

| □ No, it didn’t | □ Yes, it did |
| --- | --- |

**e460bis.** How did it influence you?

| □ Barrier | □ Facilitator |
| --- | --- |

**e460tris.** Why do you think this category was a barrier or facilitator?

_____________________________________________________________________________________________________________________________________________________________________________________________________________________________________________________________________

**e570. Social security services, systems and policies:** services, systems and policies aimed at providing income support to people who, because of age, poverty, unemployment, health condition or disability, require public assistance that is funded either by general tax revenues or contributory schemes.

| □ No, it didn’t | □ Yes, it did |
| --- | --- |

**e570bis.** How did it affect you?

| □ Barrier | □ Facilitator |
| --- | --- |

**e570tris.** Why do you think this category was a barrier or facilitator?

_____________________________________________________________________________________________________________________________________________________________________________________________________________________________________________________________________

**e580. Health services, systems and policies:** services, systems and policies for preventing and treating health problems, providing medical rehabilitation and promoting a healthy lifestyle.

| □ No, it didn’t | □ Yes, it did |
| --- | --- |

**e580bis.** How did it affect you?

| □ Barrier | □ Facilitator |
| --- | --- |

**e580tris.** Why do you think this category was a barrier or facilitator?

_____________________________________________________________________________________________________________________________________________________________________________________________________________________________________________________________________

**e590. Labour and employment services, systems and policies:** services, systems, and policies related to finding suitable work for persons who are unemployed or looking for different work, or to support individuals already employed who are seeking promotion.

| □ No, it didn’t | □ Yes, it did |
| --- | --- |

**e590bis.** How did it affect you?

| □ Barrier | □ Facilitator |
| --- | --- |

**e590tris.** Why do you think this category was a barrier or facilitator?

_____________________________________________________________________________________________________________________________________________________________________________________________________________________________________________________________________

**23. In your opinion, were there any questions that were difficult to understand?**

□ No

□ Yes

23bis. Which questions?

_____________________________________________________________________________________________________________________________________________________________________________________________________________________________________________________________________

**24. Do you think the length of the questionnaire is adequate?**

□ No

□ Yes

24bis. Do you have any suggestions?: _____________________________________________________________________________________________________________________________________________________________________________________________________________________________________________________________________

**25. Do you think there are any relevant aspects that were not investigated by the questionnaire?**

□ No

□ Yes

25bis. Which aspects?

_____________________________________________________________________________________________________________________________________________________________________________________________________________________________________________________________________

**26. Duration of the interview: _____________ minutes**

**27. Did the patient need help answering the questions?**

□ No

□ Yes

**27bis. For which categories did he/she need support?**

_____________________________________________________________________________________________________________________________________________________________________________________________________________________________________________________________________
