# Supplementary material for: Validating the Core Set for Vocational Rehabilitation in a Population of Cancer Survivors: A Cross-Sectional Study
Source: J Occup Rehabil. 2024 Dec 11;35(4):910–28. doi: 10.1007/s10926-024-10252-5 (PMC12575594; doi:10.1007/s10926-024-10252-5)
Supplement: Supplementary file 2 — Supplementary file2 (DOCX 22 KB) [file 10926_2024_10252_MOESM2_ESM.docx]

| **Supplementary Information 2.** Type of accommodations by groups of participant | | | | | | | | |
| --- | --- | --- | --- | --- | --- | --- | --- | --- |
|  | | **Total** | **Group 1** | **Group 2** | **Group 3** | **Group 2+3** | **Group CT** | **Group NoCT** |
|  |  | n (%) | n (%) | n (%) | n (%) | n (%) | n (%) | n (%) |
|  |  | 104 | 35 (33.7) | 54 (51.9) | 15 (14.4) | 69 (66.3) | 40 (38.5) | 64 (61.5) |
| **Accommodations** | no | 58 (55.8) | 13 (22.4) | 30 (51.7) | 15 (25.9) | 45 (77.6) | 8 (13.8) | 48 (82.8) |
|  | yes | 46 (44.2) | 22 (47.8) | 24 (52.2) | 0 (0.0) | 24 (52.2) | 32 (69.6) | 16 (34.8) |
|  | *more flexibility* | 27 (59) | 12 (44.4) | 15 (55.6) | 0 (0.0) | 15 (55.6) | 19 (70.4) | 8 (29.6) |
|  | *work schedule reduction* | 15 (32.6) | 8 (53.3) | 7 (46.7) | 0 (0.0) | 7 (46.7) | 12 (80.0) | 3 (20.0) |
|  | *change of work tasks* | 18 (39.1) | 10 (55.6) | 8 (44.4) | 0 (0.0) | 8 (44.4) | 10 (55.6) | 8 (44.4) |
|  | *elimination of shifts* | 2 (4.3) | 2 (100.0) | 0 (0.0) | 0 (0.0) | 0 (0.0) | 1 (50.0) | 1 (50.0) |
|  | *transferred to the workplace nearest home* | 1 (2.2) | 1 (100.0) | 0 (0.0) | 0 (0.0) | 0 (0.0) | 1 (100.0) | 0 (0.0) |
|  | *other* | 2 (4.3) | 1 (50.0) | 1 (50.0) | 0 (0.0) | 1 (50.0) | 1 (50.0) | 1 (50.0) |
