# Supplementary material for: Validating the Core Set for Vocational Rehabilitation in a Population of Cancer Survivors: A Cross-Sectional Study
Source: J Occup Rehabil. 2024 Dec 11;35(4):910–28. doi: 10.1007/s10926-024-10252-5 (PMC12575594; doi:10.1007/s10926-024-10252-5)
Supplement: Supplementary file 3 — Supplementary file3 (DOC 63 KB) [file 10926_2024_10252_MOESM3_ESM.doc]

| **Supplementary Information 3**. Descriptive analysis of the categories classified by three groups of participants and the components BF, AP, and EF | | | | | | | | | | | | | | | | | | |
| --- | --- | --- | --- | --- | --- | --- | --- | --- | --- | --- | --- | --- | --- | --- | --- | --- | --- | --- |
|  | **Group 1 (n=35)** | | | | | | **Group 2 (n=54)** | | | | | | **Group 3 (n=15)** | | | | | |
| **Components** | **Body function (26 categories)** | **Activities and participation (33 categories)** | **Environmental factors (26 categories)** | | | | **Body function (26 categories)** | **Activities and participation (33 categories)** | **Environmental factors (26 categories)** | | | | **Body function (26 categories)** | **Activities and participation (33 categories)** | **Environmental factors (26 categories)** | | | |
|  |  |  | Yes | Barrier | Facilitator | Mixed |  |  | Yes | Barrier | Facilitator | Mixed |  |  | Yes, it has influenced | Barrier | Facilitator | Mixed |
| *Total number of categories reported (n) ** | 256 | 208 | 242 | 64 | 172 | 6 | 221 | 110 | 207 | 45 | 154 | 8 | 0 | 0 | 27 | 2 | 25 | 0 |
| *Total percentage of categories (%)* | 28.1 | 18.0 | 26.6 | 26.4 | 71.1 | 2.5 | 15.7 | 6.2 | 14.7 | 21.7 | 74.4 | 3.9 | 0 | 0 | 6.9 | 7.4 | 92.6 | 0 |
| *Range of categories (n)* | 0-14 | 0-15 | 2-15 | NA | NA | NA | 0-16 | 0-9 | 0-15 | NA | NA | NA | 0 | 0 | 0-8 | NA | NA | NA |
| *Average n. of categories (SD)* | 7.3 (3.6) | 5.9 (4.1) | 6.9 (3.1) | NA | NA | NA | 4.1 (3.7) | 2.0 (1.9) | 3.8 (3.1) | NA | NA | NA | 0 | 0 | 1.8 (2.2) | NA | NA | NA |
| Number of participants with n. categories | | | | |  |  |  |  |  |  |  |  |  |  |  |  |  |  |
| 0 categories | 1 participant | 1 | 0 | NA | NA | NA | 6 | 11 | 5 | NA | NA | NA | 0 | 0 | 6 | NA | NA | NA |
| 1 | 0 | 3 | 0 | NA | NA | NA | 11 | 17 | 6 | NA | NA | NA | 0 | 0 | 2 | NA | NA | NA |
| 2 | 2 | 3 | 1 | NA | NA | NA | 5 | 6 | 11 | NA | NA | NA | 0 | 0 | 3 | NA | NA | NA |
| 3 | 2 | 5 | 3 | NA | NA | NA | 7 | 9 | 6 | NA | NA | NA | 0 | 0 | 1 | NA | NA | NA |
| 4 | 4 | 3 | 5 | NA | NA | NA | 3 | 6 | 8 | NA | NA | NA | 0 | 0 | 2 | NA | NA | NA |
| ≥ 5 | 26 | 20 | 26 | NA | NA | NA | 22 | 5 | 18 | NA | NA | NA | 0 | 0 | 1 | NA | NA | NA |
| BF: Body functions, AP: Activities and participation, EF: Environmental factors, NA: Not applicable  Group 1= participants who perceived RTW-related difficulties, which were described through the CS-VR-Onco.  Group 2= participants who reported no perceived RTW-related difficulties, although they reported problems during this process described through the CS-VR-Onco.  Group 3= participants who did not perceive any RTW-related difficulties and did not report any problems during this process as described in the CS-VR-Onco.  * The number of categories multiplied by the number of participants gives the number and the related percentage out of the total number of possible answers | | | | | | | | | | | | | | | | | | |
