# Supplementary material for: Validating the Core Set for Vocational Rehabilitation in a Population of Cancer Survivors: A Cross-Sectional Study
Source: J Occup Rehabil. 2024 Dec 11;35(4):910–28. doi: 10.1007/s10926-024-10252-5 (PMC12575594; doi:10.1007/s10926-024-10252-5)
Supplement: Supplementary file 4 — Supplementary file4 (DOC 63 KB) [file 10926_2024_10252_MOESM4_ESM.doc]

| **Supplementary Information 4.** Descriptive analysis of the categories classified by two groups of participants and the chapters of the BF and AP components | | | | | | | | | | | | | | | | | | | | | | | | | |
| --- | --- | --- | --- | --- | --- | --- | --- | --- | --- | --- | --- | --- | --- | --- | --- | --- | --- | --- | --- | --- | --- | --- | --- | --- | --- |
| **Components** | | **Body function** | | | | | | | | **Activities and participation** | | | | | | | | | | | | | | | |
| **Chapters** | | **b1. Mental functions**  **(9 categories)** | | **b2. Sensory functions and pain**  **(6 categories)** | | **b4. Exercise and tolerance functions**  **(2 categories)** | | **Other functions**  **(9 categories)** | | **d1. Learning and applying knowledge**  **(5 categories)** | | **d2. General tasks and demands**  **(4 categories)** | | **d3. Communication**  **(2 categories)** | | **d4. Mobility**  **(10 categories)** | | **d5. Self-care**  **(4 categories)** | | **d7. Interpersonal interactions and relationships**  **(5 categories)** | | **d8. Major life areas**  **(2 categories)** | | **d9. Community, social and civic life**  **(1 category)** | |
|  | | Group 1 (n=35) | Group 2 + 3 (n=69) | Group 1 (n=35) | Group 2 + 3 (n=69) | Group 1 (n=35) | Group 2 + 3 (n=69) | Group 1 (n=35) | Group 2 + 3 (n=69) | Group 1 (n=35) | Group 2 + 3 (n=69) | Group 1 (n=35) | Group 2 + 3 (n=69) | Group 1 (n=35) | Group 2 + 3 (n=69) | Group 1 (n=35) | Group 2 + 3 (n=69) | Group 1 (n=35) | Group 2 + 3 (n=69) | Group 1 (n=35) | Group 2 + 3 (n=69) | Group 1 (n=35) | Group 2 + 3 (n=69) | Group 1 (n=35) | Group 2 + 3 (n=69) |
| *Total number of categories reported (n) ** | | 120 | 103 | 42 | 30 | 33 | 30 | 61 | 58 | 29 | 15 | 42 | 26 | 2 | 2 | 34 | 46 | 7 | 1 | 36 | 10 | 16 | 7 | 6 | 3 |
| *Total percentage of categories (%)* | | 38.1 | 16.6 | 20.0 | 7.2 | 47.1 | 21.7 | 19.4 | 9.3 | 16.6 | 4.3 | 30.0 | 9.4 | 2.9 | 1.4 | 9.7 | 6.7 | 5.0 | 0.4 | 20.6 | 2.9 | 22.9 | 5.1 | 17.1 | 4.3 |
| *Range of categories (n)* | | 0-8 | 0-5 | 0-4 | 0-5 | 0-2 | 0-2 | 0-5 | 0-6 | 0-4 | 0-2 | 0-4 | 0-3 | 0-2 | 0-1 | 0-8 | 0-5 | 0-3 | 0-1 | 0-5 | 0-2 | 0-2 | 0-2 | 0-1 | 0-1 |
| *Average of categories (SD)* | | 3.4 (2.2) | 1.5 (1.7) | 1.2 (1.1) | 0.4 (0.9) | 0.9 (0.4) | 0.4 (0.6) | 1.7 (1.3) | 0.8 (1.3) | 0.8 (1.0) | 0.2 (0.5) | 1.2 (1.1) | 0.4 (0.7) | 0.1 (0.3) | 0.0 (0.2) | 2.0 (2.0) | 0.7 (1.1) | 0.2 (0.6) | 0.0 (0.1) | 1.0 (1.4) | 0.1 (0.4) | 0.5 (0.6) | 0.1 (0.4) | 0.2 (0.4) | 0.0 (0.2) |
| Number of participants with n. categories | | | | | | |  |  |  |  |  |  |  |  |  |  |  |  |  |  |  |  |  |  |  |
| 0 categories | 3 participants | | 28 | 11 | 49 | 4 | 41 | 6 | 41 | 17 | 57 | 10 | 50 | 34 | 67 | 9 | 43 | 30 | 68 | 18 | 61 | 21 | 63 | 29 | 66 |
| 1 | 5 | | 14 | 11 | 14 | 29 | 26 | 12 | 13 | 10 | 9 | 15 | 13 | 0 | 2 | 10 | 14 | 4 | 1 | 7 | 6 | 12 | 5 | 6 | 3 |
| 2 | 4 | | 9 | 9 | 4 | 2 | 2 | 7 | 7 | 6 | 3 | 4 | 5 | 1 | 0 | 4 | 7 | 0 | 0 | 4 | 2 | 2 | 1 | 0 | 0 |
| 3 | 8 | | 7 | 3 | 1 | 0 | 0 | 6 | 4 | 1 | 0 | 5 | 1 | 0 | 0 | 4 | 3 | 5 | 0 | 4 | 0 | 0 | 0 | 0 | 0 |
| 4 | 3 | | 5 | 1 | 0 | 0 | 0 | 3 | 2 | 1 | 0 | 1 | 0 | 0 | 0 | 5 | 1 | 0 | 0 | 1 | 0 | 0 | 0 | 0 | 0 |
| ≥ 5 | 12 | | 6 | 0 | 1 | 0 | 0 | 1 | 2 | 0 | 0 | 0 | 0 | 0 | 0 | 3 | 1 | 0 | 0 | 1 | 0 | 0 | 0 | 0 | 0 |
| BF: Body functions, AP: Activities and participation  Group 1= participants who perceived RTW-related difficulties, which were described through the CS-VR-Onco.  Group 2+3= participants who reported no perceived RTW-related difficulties but who reported problems described through the CS-VR-Onco or did not report problems.  * The number of categories multiplied by the number of participants gives the number and the related percentage out of the total number of possible answers. | | | | | | | | | | | | | | | | | | | | | | | | | |
