# Supplementary material for: Validating the Core Set for Vocational Rehabilitation in a Population of Cancer Survivors: A Cross-Sectional Study
Source: J Occup Rehabil. 2024 Dec 11;35(4):910–28. doi: 10.1007/s10926-024-10252-5 (PMC12575594; doi:10.1007/s10926-024-10252-5)
Supplement: Supplementary file 5 — Supplementary file5 (DOC 113 KB) [file 10926_2024_10252_MOESM5_ESM.doc]

| **Supplementary Information 5**. Descriptive analysis of the categories classified by two groups of participants and the chapters of the EF component | | | | | | | | | | | | | | | | | | | | | | | | | | | | | | | |
| --- | --- | --- | --- | --- | --- | --- | --- | --- | --- | --- | --- | --- | --- | --- | --- | --- | --- | --- | --- | --- | --- | --- | --- | --- | --- | --- | --- | --- | --- | --- | --- |
| **Component** | **Environmental factors** | | | | | | | | | | | | | | | | | | | | | | | | | | | | | | |
| **Chapters** | **e1. Products and technology (4 categories)** | | | | | | | | | | | **e2. Natural environment and human-made changes to environment (4 categories)** | | | | | | | | | | **e3. Support and relationships (8 categories)** | | | | | | | | | |
|  | **Group 1 (n=35)** | | | | | | **Group 2 + 3 (n=69)** | | | | | **Group 1 (n=35)** | | | | | **Group 2 + 3 (n=69)** | | | | | **Group 1 (n=35)** | | | | | **Group 2 + 3 (n=69)** | | | | |
|  | **No*** | **Yes**** | | **Barrier** | **Facilitator** | **Mixed** | **No** | **Yes** | **Barrier** | **Facilitator** | **Mixed** | **No** | **Yes** | **Barrier** | **Facilitator** | **Mixed** | **No** | **Yes** | **Barrier** | **Facilitator** | **Mixed** | **No** | **Yes** | **Barrier** | **Facilitator** | **Mixed** | **No** | **Yes** | **Barrier** | **Facilitator** | **Mixed** |
| *Total number of categories reported (n)**** | 93 | 47 | | 19 | 25 | 3 | 240 | 36 | 17 | 17 | 2 | 127 | 13 | 11 | 2 | 0 | 206 | 11 | 10 | 1 | 0 | 162 | 118 | 13 | 103 | 2 | 422 | 130 | 5 | 122 | 3 |
| *Total percentage of categories (%)* | 66.4 | 33.6 | | 40.4 | 53.2 | 6.4 | 87.0 | 13.0 | 47.2 | 47.2 | 5.6 | 90.7 | 9.3 | 84.6 | 15.4 | 0.0 | 74.7 | 4.0 | 90.9 | 9.1 | 0.0 | 57.9 | 42.1 | 11.0 | 87.3 | 1.7 | 76.5 | 23.5 | 3.8 | 93.8 | 2.3 |
| *Range of categories (n)* | NA | 0-4 | | NA | NA | NA | NA | 0-4 | NA | NA | NA | NA | 0-3 | NA | NA | NA | NA | 0-2 | NA | NA | NA | NA | 0-6 | NA | NA | NA | NA | 0-6 | NA | NA | NA |
| *Average of categories (SD)* | NA | 1.3 (1.1) | | NA | NA | NA | NA | 0.5 (0.8) | NA | NA | NA | NA | 0.4 (0.7) | NA | NA | NA | NA | 0.2 (0.4) | NA | NA | NA | NA | 3.4 (1.5) | NA | NA | NA | NA | 1.9 (1.5) | NA | NA | NA |
| Number of participants with n. categories | | | | | | | | | | | |  |  |  |  |  |  |  |  |  |  |  |  |  |  |  |  |  |  |  |  |
| 0 categories | NA | | 8 participants | NA | NA | NA | NA | 43 | NA | NA | NA | NA | 26 | NA | NA | NA | NA | 59 | NA | NA | NA | NA | 1 | NA | NA | NA | NA | 17 | NA | NA | NA |
| 1 | NA | | 14 | NA | NA | NA | NA | 20 | NA | NA | NA | NA | 6 | NA | NA | NA | NA | 9 | NA | NA | NA | NA | 2 | NA | NA | NA | NA | 11 | NA | NA | NA |
| 2 | NA | | 7 | NA | NA | NA | NA | 3 | NA | NA | NA | NA | 2 | NA | NA | NA | NA | 1 | NA | NA | NA | NA | 8 | NA | NA | NA | NA | 21 | NA | NA | NA |
| 3 | NA | | 5 | NA | NA | NA | NA | 2 | NA | NA | NA | NA | 1 | NA | NA | NA | NA | 0 | NA | NA | NA | NA | 6 | NA | NA | NA | NA | 7 | NA | NA | NA |
| 4 | NA | | 1 | NA | NA | NA | NA | 1 | NA | NA | NA | NA | 0 | NA | NA | NA | NA | 0 | NA | NA | NA | NA | 10 | NA | NA | NA | NA | 10 | NA | NA | NA |
| ≥ 5 | NA | | 0 | NA | NA | NA | NA | 0 | NA | NA | NA | NA | 0 | NA | NA | NA | NA | 0 | NA | NA | NA | NA | 8 | NA | NA | NA | NA | 3 | NA | NA | NA |
| EF: Environmental factors. NA: Not applicable  * No: the number of categories that did not affect CS  ** Yes: the number of categories that did affect CS  *** The number of categories multiplied by the number of participants gives the number and the related percentage out of the total number of possible answers | | | | | | | | | | | | | | | | | | | | | | | | | | | | | |  |  |

| **Supplementary Information 5**. Descriptive analysis of the factors classified by two groups of participants and the chapters of the EF component | | | | | | | | | | | | | | | | | | | | | | |
| --- | --- | --- | --- | --- | --- | --- | --- | --- | --- | --- | --- | --- | --- | --- | --- | --- | --- | --- | --- | --- | --- | --- |
| **Component** | | **Environmental factors** | | | | | | | | | | | | | | | | | | | | |
| **Chapters** | **e4. Attitudes (7 categories)** | | | | | | | | | | | | **e5. Services. systems and policies (3 categories)** | | | | | | | | | |
|  | **Group 1 (n=35)** | | | | | | | **Group 2 + 3 (n=69)** | | | | | **Group 1 (n=35)** | | | | | **Group 2 + 3 (n=69)** | | | | |
|  | **No*** | | **Yes**** | | **Barrier** | **Facilitator** | **Mixed** | **No** | **Yes** | **Barrier** | **Facilitator** | **Mixed** | **No** | **Yes** | **Barrier** | **Facilitator** | **Mixed** | **No** | **Yes** | **Barrier** | **Facilitator** | **Mixed** |
| *Total number of factors reported (n)**** | 207 | | 38 | | 17 | 20 | 1 | 444 | 39 | 9 | 27 | 3 | 79 | 26 | 5 | 22 | 0 | 189 | 18 | 6 | 12 | 0 |
| *Total percentage of factors (%)* | 84.5 | | 15.5 | | 44.7 | 52.6 | 2.6 | 91.9 | 8.1 | 23.1 | 69.2 | 7.7 | 75.2 | 24.8 | 19.2 | 84.6 | 0.0 | 91.3 | 8.7 | 33.3 | 66.7 | 0.0 |
| *Range of factors (n)* | NA | | 0-5 | | NA | NA | NA | NA | 0-4 | NA | NA | NA | NA | 0-3 | NA | NA | NA | NA | 0-2 | NA | NA | NA |
| *Average of factors (SD* | NA | | 1.1 (1.4) | | NA | NA | NA | NA | 0.6 (1.0) | NA | NA | NA | NA | 0.7 (0.9) | NA | NA | NA | NA | 0.3 (0.6) | NA | NA | NA |
| Distribution of the frequency of factors | | | | | | | | | | | | | | | | | | | | | | |
| 0 factors | NA | | | 17 | NA | NA | NA | NA | 46 | NA | NA | NA | NA | 17 | NA | NA | NA | NA | 56 | NA | NA | NA |
| 1 | NA | | | 9 | NA | NA | NA | NA | 15 | NA | NA | NA | NA | 11 | NA | NA | NA | NA | 8 | NA | NA | NA |
| 2 | NA | | | 3 | NA | NA | NA | NA | 2 | NA | NA | NA | NA | 6 | NA | NA | NA | NA | 5 | NA | NA | NA |
| 3 | NA | | | 2 | NA | NA | NA | NA | 4 | NA | NA | NA | NA | 1 | NA | NA | NA | NA | 0 | NA | NA | NA |
| 4 | NA | | | 3 | NA | NA | NA | NA | 2 | NA | NA | NA | NA | 0 | NA | NA | NA | NA | 0 | NA | NA | NA |
| ≥ 5 | NA | | | 1 | NA | NA | NA | NA | 0 | NA | NA | NA | NA | 0 | NA | NA | NA | NA | 0 | NA | NA | NA |
| EF: Environmental factors. NA: Not applicable  * No, the factor did not affect CS  ** Yes, the factor did affect CS  *** The number of categories multiplied by the number of participants gives the number and the related percentage out of the total number of possible answers | | | | | | | | | | | | | | | | | | | | | | |
