# Supplementary material for: Validating the Core Set for Vocational Rehabilitation in a Population of Cancer Survivors: A Cross-Sectional Study
Source: J Occup Rehabil. 2024 Dec 11;35(4):910–28. doi: 10.1007/s10926-024-10252-5 (PMC12575594; doi:10.1007/s10926-024-10252-5)
Supplement: Supplementary file 6 — Supplementary file6 (DOCX 31 KB) [file 10926_2024_10252_MOESM6_ESM.docx]

| **Supplementary Information 6.** Descriptive analysis of the categories classifieds by treatment (chemotherapy) and the chapters of the BF and AP components | | | | | | | | | | | | | | | | | | | | | | | | |
| --- | --- | --- | --- | --- | --- | --- | --- | --- | --- | --- | --- | --- | --- | --- | --- | --- | --- | --- | --- | --- | --- | --- | --- | --- |
| **Components** | **Body function** | | | | | | | | **Activities and participation** | | | | | | | | | | | | | | | |
| **Chapters** | **b1. Mental functions (9 categories)** | | **b2. Sensory functions and pain (6 categories)** | | **b4. Exercise and tolerance functions (2 categories)** | | **Other functions (9 categories)** | | **d1. Learning and applying knowledge (5 categories)** | | **d2. General tasks and demands (4 categories)** | | **d3. Communication (2 categories)** | | **d4. Mobility (10 categories)** | | **d5. Self-care (4 categories)** | | **d7. Interpersonal interactions and relationships (5 categories)** | | **d8. Major life areas (2 categories)** | | **d9. Community, social, and civic life (1 category)** | |
|  | **Group CT (n=40)** | **Group NoCT (n=64)** | **Group CT (n=40)** | **Group NoCT (n=64)** | **Group CT (n=40)** | **Group NoCT (n=64)** | **Group CT (n=40)** | **Group NoCT (n=64)** | **Group CT (n=40)** | **Group NoCT (n=64)** | **Group CT (n=40)** | **Group NoCT (n=64)** | **Group CT (n=40)** | **Group NoCT (n=64)** | **Group CT (n=40)** | **Group NoCT (n=64)** | **Group CT (n=40)** | **Group NoCT (n=64)** | **Group CT (n=40)** | **Group NoCT (n=64)** | **Group CT (n=40)** | **Group NoCT (n=64)** | **Group CT (n=40)** | **Group NoCT (n=64)** |
| *Total number of categories reported (n)** | 100 | 123 | 42 | 30 | 39 | 24 | 68 | 51 | 20 | 24 | 29 | 39 | 1 | 3 | 58 | 58 | 1 | 7 | 15 | 31 | 9 | 14 | 3 | 6 |
| *Total percentage of categories (%)* | 27.8 | 21.4 | 17.5 | 7.8 | 48.8 | 18.8 | 18.9 | 8.9 | 10.0 | 7.5 | 18.1 | 15.2 | 1.25 | 2.3 | 14.5 | 9.1 | 0.6 | 2.7 | 7.5 | 9.7 | 11.2 | 11.0 | 7.5 | 9.4 |
| *Range of categories (n)* | 0-5 | 0-8 | 0-5 | 0-4 | 0-2 | 0-2 | 0-6 | 0-3 | 0-4 | 0-3 | 0-3 | 0-4 | 0-1 | 0-2 | 0-8 | 0-4 | 0-1 | 0-3 | 0-3 | 0-5 | 0-2 | 0-2 | 0-1 | 0-1 |
| *Average n. of categories (SD)* | 2.5 (1.7) | 1.9 (2.2) | 1.1 (1.2) | 0.5 (0.8) | 1.0 (0.4) | 0.4 (0.5) | 1.70 (1.65) | 0.8 (1.1) | 0.5 (0.9) | 0.4 (0.7) | 0.7 (0.8) | 0.6 (1.0) | 0.0 (0.2) | 0.1 (0.3) | 1.5 (1.9) | 0.9 (1.3) | 0.0 (0.2) | 0.1 (0.4) | 0.4 (0.7) | 0.5 (1.1) | 0.2 (0.5) | 0.2 (0.6) | 0.1 (0.3) | 0.1 (0.3) |
| Number of participants with n. categories | | | | | | | | | | | | | | | | | | | | | | | | |
| *0 categories* | 6 participants | 25 | 16 | 44 | 4 | 41 | 10 | 37 | 27 | 47 | 18 | 42 | 39 | 62 | 15 | 37 | 39 | 59 | 29 | 50 | 32 | 52 | 37 | 58 |
| *1* | 8 | 11 | 12 | 13 | 33 | 22 | 14 | 11 | 8 | 11 | 17 | 11 | 1 | 1 | 12 | 12 | 1 | 4 | 8 | 5 | 7 | 10 | 3 | 6 |
| *2* | 5 | 8 | 8 | 5 | 3 | 1 | 6 | 8 | 4 | 5 | 3 | 6 | 0 | 1 | 6 | 5 | 0 | 0 | 2 | 4 | 1 | 2 | 0 | 0 |
| *3* | 10 | 5 | 3 | 1 | 0 | 0 | 2 | 8 | 0 | 1 | 2 | 4 | 0 | 0 | 3 | 4 | 0 | 1 | 1 | 3 | 0 | 0 | 0 | 0 |
| *4* | 3 | 5 | 0 | 1 | 0 | 0 | 5 | 0 | 1 | 0 | 0 | 1 | 0 | 0 | 0 | 6 | 0 | 0 | 0 | 1 | 0 | 0 | 0 | 0 |
| *≥ 5* | 8 | 10 | 1 | 0 | 0 | 0 | 3 | 0 | 0 | 0 | 0 | 0 | 0 | 0 | 4 | 0 | 0 | 0 | 0 | 1 | 0 | 0 | 0 | 0 |
| BF: Body functions, AP: Activities and participation  Group CT= patients who had undergone chemotherapy  Group NoCT= patients who had not undergone chemotherapy  * The number of categories multiplied by the number of participants gives the number and the related percentage out of the total number of possible answers | | | | | | | | | | | | | | | | | | | | | | | | |
