# Supplementary material for: Validating the Core Set for Vocational Rehabilitation in a Population of Cancer Survivors: A Cross-Sectional Study
Source: J Occup Rehabil. 2024 Dec 11;35(4):910–28. doi: 10.1007/s10926-024-10252-5 (PMC12575594; doi:10.1007/s10926-024-10252-5)
Supplement: Supplementary file 7 — Supplementary file7 (DOCX 45 KB) [file 10926_2024_10252_MOESM7_ESM.docx]

| **Supplementary Information 7.** Descriptive analysis of the problems categorized by treatment (chemotherapy) and the chapters of the EF component | | | | | | | | | | | | | | | | | | | | | | | | | | | | | | |
| --- | --- | --- | --- | --- | --- | --- | --- | --- | --- | --- | --- | --- | --- | --- | --- | --- | --- | --- | --- | --- | --- | --- | --- | --- | --- | --- | --- | --- | --- | --- |
| **Components** | **Environmental factors** | | | | | | | | | | | | | | | | | | | | | | | | | | | | | |
| **Chapters** | **e1. Products and technology (4 categories)** | | | | | | | | | | **e2. Natural environment and human-made changes to environment (4 categories)** | | | | | | | | | | **e3. Support and relationships (8 categories)** | | | | | | | | | |
|  | **Group CT (n=40)** | | | | | **Group NoCT (n=64)** | | | | | **Group CT (n=40)** | | | | | **Group NoCT (n=64)** | | | | | **Group CT (n=40)** | | | | | **Group NoCT (n=64)** | | | | |
|  | **No*** | **Yes**** | **Barrier** | **Facilitator** | **Mixed** | **No*** | **Yes**** | **Barrier** | **Facilitator** | **Mixed** | **No*** | **Yes**** | **Barrier** | **Facilitator** | **Mixed** | **No*** | **Yes**** | **Barrier** | **Facilitator** | **Mixed** | **No*** | **Yes**** | **Barrier** | **Facilitator** | **Mixed** | **No*** | **Yes**** | **Barrier** | **Facilitator** | **Mixed** |
| *Total number of answers (n)* | **160** | **160** | **47** | **47** | **47** | **256** | **256** | **36** | **36** | **36** | **160** | **160** | **12** | **12** | **12** | **256** | **256** | **12** | **12** | **12** | **320** | **320** | **131** | **131** | **131** | **512** | **512** | **117** | **117** | **117** |
| *Total number of problems detected (n)* | 113 | 47 | 25 | 18 | 4 | 220 | 36 | 11 | 24 | 1 | 148 | 12 | 11 | 1 | 0 | 244 | 12 | 10 | 2 | 0 | 189 | 131 | 12 | 115 | 4 | 395 | 117 | 6 | 110 | 1 |
| *Total percentage of problems (%)* | 70.6 | 29.4 | 53.2 | 38.3 | 8.5 | 85.9 | 14.1 | 30.6 | 66.7 | 2.8 | 92.5 | 7.5 | 91.7 | 8.3 | 0.0 | 95.3 | 4.7 | 83.3 | 16.7 | 0.0 | 59.1 | 40.9 | 9.2 | 87.8 | 3.1 | 77.1 | 22.9 | 5.1 | 94.0 | 0.9 |
| *Range of problems (n)* | NA | 0-4 | NA | NA | NA | NA | 0-2 | NA | NA | NA | NA | 0-2 | NA | NA | NA | NA | 0-1 | NA | NA | NA | NA | 0-6 | NA | NA | NA | NA | 0-5 | NA | NA | NA |
| *Average n. of problems (SD)* | NA | 1.2 (1.2) | NA | NA | NA | NA | 0.6 (0.8) | NA | NA | NA | NA | 0.3 (0.6) | NA | NA | NA | NA | 0.2 (0.5) | NA | NA | NA | NA | 3.3 (1.6) | NA | NA | NA | NA | 1.8 (1.5) | NA | NA | NA |
| **Number of participants with n. problems** |  |  |  |  |  |  |  |  |  |  |  |  |  |  |  |  |  |  |  |  |  |  |  |  |  |  |  |  |  |  |
| *0 problems* | NA | 13 | NA | NA | NA | NA | 38 | NA | NA | NA | NA | 31 | NA | NA | NA | NA | 54 | NA | NA | NA | NA | 1 | NA | NA | NA | NA | 17 | NA | NA | NA |
| *1* | NA | 16 | NA | NA | NA | NA | 18 | NA | NA | NA | NA | 6 | NA | NA | NA | NA | 9 | NA | NA | NA | NA | 3 | NA | NA | NA | NA | 10 | NA | NA | NA |
| *2* | NA | 4 | NA | NA | NA | NA | 6 | NA | NA | NA | NA | 3 | NA | NA | NA | NA | 0 | NA | NA | NA | NA | 12 | NA | NA | NA | NA | 17 | NA | NA | NA |
| *3* | NA | 5 | NA | NA | NA | NA | 2 | NA | NA | NA | NA | 0 | NA | NA | NA | NA | 1 | NA | NA | NA | NA | 5 | NA | NA | NA | NA | 8 | NA | NA | NA |
| *4* | NA | 2 | NA | NA | NA | NA | 0 | NA | NA | NA | NA | 0 | NA | NA | NA | NA | 0 | NA | NA | NA | NA | 9 | NA | NA | NA | NA | 11 | NA | NA | NA |
| *≥5* | NA | 0 | NA | NA | NA | NA | 0 | NA | NA | NA | NA | 0 | NA | NA | NA | NA | 0 | NA | NA | NA | NA | 10 | NA | NA | NA | NA | 1 | NA | NA | NA |
| BF: Body functions, AP: Activities and participation Group CT= patients who had undergone chemotherapy Group NoCT= patients who had not undergone chemotherapy | | | | | | | | | | | | | | | | | | | | | | | | | | | | | | |

| **Supplementary material 7**. Descriptive analysis of the problems categorized by treatment (chemotherapy) and the chapters of the EF component | | | | | | | | | | | | | | | | | | | | |
| --- | --- | --- | --- | --- | --- | --- | --- | --- | --- | --- | --- | --- | --- | --- | --- | --- | --- | --- | --- | --- |
| **Components** | Environmental factors | | | | | | | | | | | | | | | | | | | |
| **Chapters** | **e4. Attitudes (7 categories)** | | | | | | | | | | **e5. Services, systems and policies (3 categories)** | | | | | | | | | |
|  | **Group CT (n=40)** | | | | | **Group NoCT (n=64)** | | | | | **Group CT (n=40)** | | | | | **Group NoCT (n=64)** | | | | |
|  | **No*** | **Yes**** | **Barrier** | **Facilitator** | **Mixed** | **No*** | **Yes**** | **Barrier** | **Facilitator** | **Mixed** | **No*** | **Yes**** | **Barrier** | **Facilitator** | **Mixed** | **No*** | **Yes**** | **Barrier** | **Facilitator** | **Mixed** |
| *Total number of answers (n)* | **280** | **280** | **42** | **42** | **42** | **448** | **448** | **35** | **35** | **35** | **120** | **120** | **28** | **28** | **28** | **192** | **192** | **16** | **16** | **16** |
| *Total number of problems detected (n)* | 238 | 42 | 16 | 23 | 3 | 413 | 35 | 10 | 24 | 1 | 91 | 28 | 5 | 23 | 0 | 176 | 16 | 6 | 10 | 0 |
| *Total percentage of problems (%)* | 85.0 | 15.0 | 38.1 | 54.8 | 7.1 | 92.2 | 7.8 | 28.6 | 68.6 | 2.9 | 75.8 | 23.3 | 17.9 | 82.1 | 0.0 | 91.7 | 8.3 | 37.5 | 62.5 | 0.0 |
| *Range of problems (n)* | NA | 0-5 | NA | NA | NA | NA | 0-4 | NA | NA | NA | NA | 0-2 | NA | NA | NA | NA | 0-3 | NA | NA | NA |
| *Average n. of problems (SD)* | NA | 1.1 (1.43) | NA | NA | NA | NA | 0.6 (1.0) | NA | NA | NA | NA | 0.7 (0.8) | NA | NA | NA | NA | 0.3 (0.6) | NA | NA | NA |
| **Number of participants with n. problems** |  |  |  |  |  |  |  |  |  |  |  |  |  |  |  |  |  |  |  |  |
| *0 problems* | NA | 20 | NA | NA | NA | NA | 43 | NA | NA | NA | NA | 21 | NA | NA | NA | NA | 52 | NA | NA | NA |
| *1* | NA | 11 | NA | NA | NA | NA | 13 | NA | NA | NA | NA | 10 | NA | NA | NA | NA | 9 | NA | NA | NA |
| *2* | NA | 1 | NA | NA | NA | NA | 4 | NA | NA | NA | NA | 9 | NA | NA | NA | NA | 2 | NA | NA | NA |
| *3* | NA | 4 | NA | NA | NA | NA | 2 | NA | NA | NA | NA | 0 | NA | NA | NA | NA | 1 | NA | NA | NA |
| *4* | NA | 3 | NA | NA | NA | NA | 2 | NA | NA | NA | NA | 0 | NA | NA | NA | NA | 0 | NA | NA | NA |
| *≥5* | NA | 1 | NA | NA | NA | NA | 0 | NA | NA | NA | NA | 0 | NA | NA | NA | NA | 0 | NA | NA | NA |
